# Oncogenic alterations in advanced NSCLC: a molecular super-highway

**DOI:** 10.1186/s40364-024-00566-0

**Published:** 2024-02-12

**Authors:** Alex Friedlaender, Maurice Perol, Giuseppe Luigi Banna, Kaushal Parikh, Alfredo Addeo

**Affiliations:** 1 Clinique Générale Beaulieu, Geneva, Switzerland; 2 Department of Medical Oncology, Centre Léon Bérard, Lyon, France; 3 Portsmouth Hospitals University NHS Trust, Portsmouth, UK; 4 Faculty of Science and Health, School of Pharmacy and Biomedical Sciences, University of Portsmouth, Portsmouth, UK; 5 Mayo Clinic, Rochester, MN USA; 6 Oncology Department, University Hospital Geneva, Rue Gentil Perret 4. 1205, Geneva, Switzerland

## Abstract

Lung cancer ranks among the most common cancers world-wide and is the first cancer-related cause of death. The classification of lung cancer has evolved tremendously over the past two decades. Today, non-small cell lung cancer (NSCLC), particularly lung adenocarcinoma, comprises a multitude of molecular oncogenic subsets that change both the prognosis and management of disease.

Since the first targeted oncogenic alteration identified in 2004, with the epidermal growth factor receptor (EGFR), there has been unprecedented progress in identifying and targeting new molecular alterations. Almost two decades of experience have allowed scientists to elucidate the biological function of oncogenic drivers and understand and often overcome the molecular basis of acquired resistance mechanisms. Today, targetable molecular alterations are identified in approximately 60% of lung adenocarcinoma patients in Western populations and 80% among Asian populations. Oncogenic drivers are largely enriched among non-smokers, east Asians, and younger patients, though each alteration has its own patient phenotype.

The current landscape of druggable molecular targets includes EGFR, anaplastic lymphoma kinase (ALK), v-raf murine sarcoma viral oncogene homolog B (BRAF), ROS proto-oncogene 1 (ROS1), Kirstin rat sarcoma virus (KRAS), human epidermal receptor 2 (HER2), c-MET proto-oncogene (MET), neurotrophic receptor tyrosine kinase (NTRK), rearranged during transfection (RET), neuregulin 1 (NRG1). In addition to these known targets, others including Phosphoinositide 3-kinases (PI3K) and fibroblast growth factor receptor (FGFR) have garnered significant attention and are the subject of numerous ongoing trials.

In this era of personalized, precision medicine, it is of paramount importance to identify known or potential oncogenic drivers in each patient. The development of targeted therapy is mirrored by diagnostic progress. Next generation sequencing offers high-throughput, speed and breadth to identify molecular alterations in entire genomes or targeted regions of DNA or RNA. It is the basis for the identification of the majority of current druggable alterations and offers a unique window into novel alterations, and de novo and acquired resistance mechanisms.

In this review, we discuss the diagnostic approach in advanced NSCLC, focusing on current oncogenic driver alterations, through their pathophysiology, management, and future perspectives. We also explore the shortcomings and hurdles encountered in this rapidly evolving field.

## Introduction

The incidence of cancer is rising across the globe, with an expected 28.4 million people living with cancer in 2040, a roughly 50% increase compared to 2020. Lung cancer ranks among the most common cancers world-wide and is the first cancer-related cause of death. Currently, patients are most often diagnosed with lung cancer at an advanced disease stage, with a poor prognosis [1, 2].

**Fig. 1:**
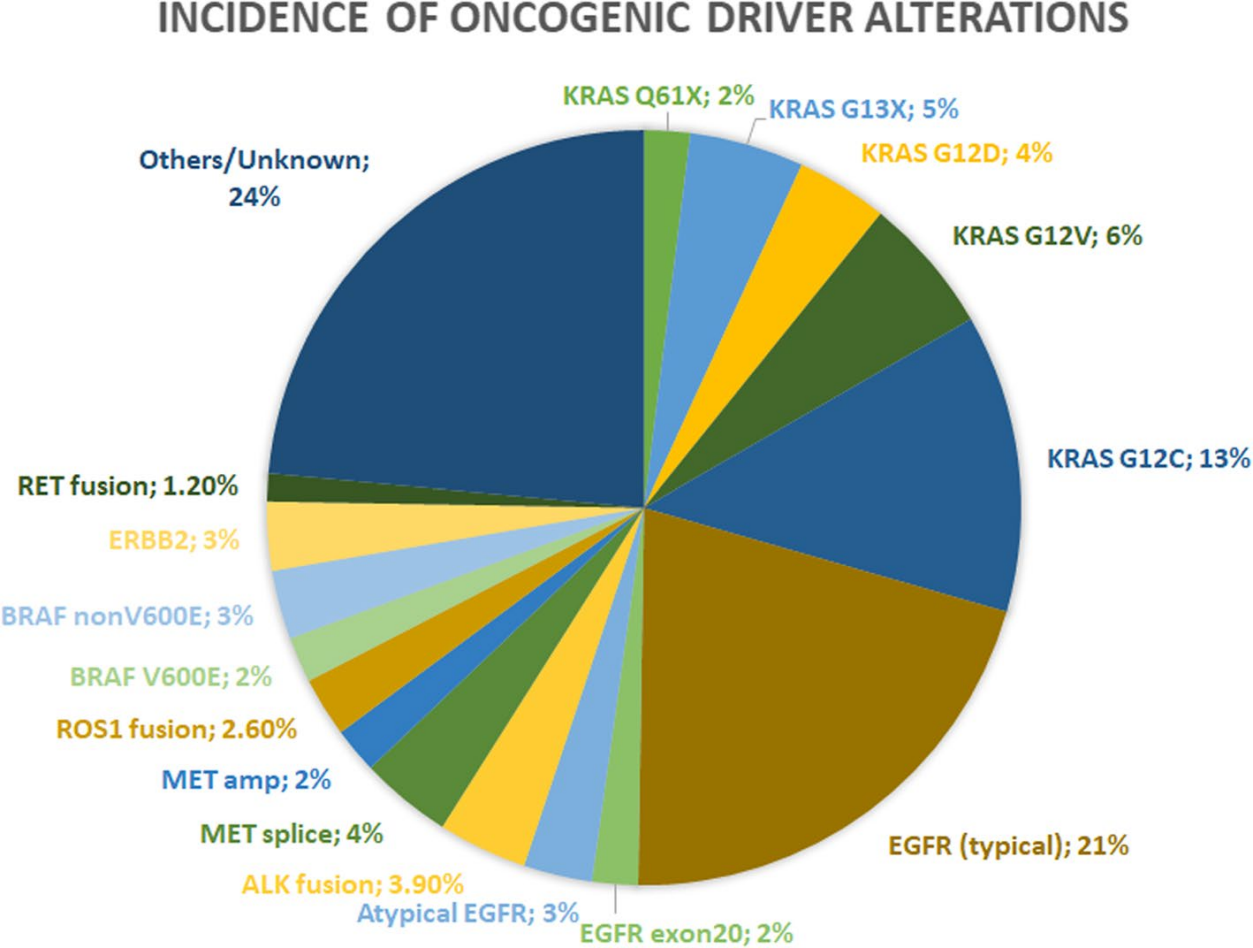
Incidence of oncogenic driver alterations in advanced non-small-cell lung adenocarcinoma. RET: rearranged during transfection, ERBB2: human epidermal growth factor receptor 2, BRAF: B-raf murine sarcoma viral homolog B, ROS1: ROS proto-oncogene 1, MET: c-Met, amp: amplification, ALK: anaplastic lymphoma kinase, EGFR: epidermal growth factor receptor, KRAS: Kirsten rat sarcoma

The classification of lung cancer has evolved tremendously, going successively from a single entity, to the distinction between small-cell and non-small cell lung cancer (NSCLC), to a histologic subdivision of the latter into squamous cell carcinoma, large cell carcinoma and adenocarcinoma. Today, lung adenocarcinoma comprises a multitude of molecular oncogenic subsets that change both the prognosis and management of disease (Fig. 1).

Over the course of the last two decades, two paradigm-shifting therapeutic developments came to light in NSCLC: the implementation of immune-checkpoint inhibitors targeting the programmed-death ligand 1 axis and the identification and targeted treatment of genomic oncogenic drivers. While the former have revolutionized therapy for patients with advanced NSCLC without oncogenic alterations, they have a lacklustre effect in all but a minority of oncogene-driven NSCLC subtypes. This is inherent to the tumour biology of oncogene-driven lung cancers, which have an immune-poor microenvironment and, due to single driving carcinogenic motors, lack the neoantigenic richness and high number of somatic mutations induced by smoking, as seen in non-oncogene-driven tumours [3].

Since the first targeted oncogenic alteration identified in 2004 [4], with the epidermal growth factor receptor (EGFR), there has been unprecedented progress in identifying and treating new molecular alterations. Almost two decades of experience have allowed scientists to elucidate the biological function of oncogenic drivers and understand and often overcome the molecular basis of acquired resistance mechanisms.

Today, targetable molecular alterations are identified in approximately 60% of lung adenocarcinoma patients in Western populations and 80% among Asian populations [5]. Oncogenic drivers are largely enriched among non-smokers, east Asians, and younger patients. This is an important consideration, as radiologic screening programmes focused on exposure to tobacco likely overlook the bulk of these patients, as they do not fit into the high-risk category. As such, oncogene-driven NSCLC are not expected to be downstaged significantly in the coming decade as no early diagnostic or screening approach is validated for this seemingly low-risk, often non-smoker population. However, there is an ongoing effort to evaluate the utility of lung cancer screening. The FANSS study of 201 participants showed feasibility of lung cancer screening in in younger females of Asian descent without any smoking history. This study had an invasive adenocarcinoma detection rate of 1.5%, comparable to TALENT study (1.5%) and superior than NLST trial (1.1%) [6].

The current landscape of druggable molecular targets includes EGFR, anaplastic lymphoma kinase (ALK), v-raf murine sarcoma viral oncogene homolog B (BRAF), ROS proto-oncogene 1 (ROS1), Kirstin rat sarcoma virus (KRAS), human epidermal receptor 2 (HER2), c-MET proto-oncogene (MET), rearranged neurotrophic receptor tyrosine kinase (NTRK), rearranged during transfection (RET), and neuregulin 1 (NRG1). In addition to these known targets, others including Phosphoinositide 3-kinase (PI3K) and fibroblast growth factor receptor (FGFR) have garnered significant attention and are the subject of numerous ongoing trials.

In this era of personalized, precision medicine, it is of paramount importance to identify known or potential oncogenic drivers in each patient. Today, Next Generation Sequencing (NGS) is synonymous with precision oncology. This massive parallel sequencing approach offers high-throughput, speed, depth and breadth to determine the order of nucleotides, or molecular alterations, in entire genomes or targeted regions of DNA or RNA. NGS is the basis for the identification of the majority of current druggable alterations and offers a unique window into novel alterations, and de novo and acquired resistance mechanisms.

In this review, we discuss the diagnostic approach in advanced NSCLC, focusing on current oncogenic driver alterations, through their pathophysiology, management, and future perspectives. We also explore the shortcomings and hurdles encountered in this rapidly evolving field.

### Diagnosis of oncogenic drivers

The identification of targetable oncogenes is essential: both to offer optimal front-line therapy and to avoid the use of costly, ineffective and potentially dangerous treatments in this subset of patients. Oncology societies such as the ESMO and ASCO [7] recommend the routine use of genomic analyses, through multiplexed assays such as NGS. The use of sequential single biomarker analyses can be less efficient in terms of turnaround time, risk of tissue attrition during tests [8] and, in many cases, cost. As a reminder, the majority of patients only have small histological or cytological samples available for analysis, as most sampling is performed through bronchoscopy or CT guided biopsies [9]. When specimens are formalin-fixed and paraffin embedded, they do not require further treatment and can be used for NGS analyses [10], and while this technique can lead to false positive or negative results from nucleic acid damage, these pre-analytical errors are rare [11]. From a cost perspective, an American analysis from 2019 suggested that sequential narrow spectrum analyses testing of 3 essential alterations, namely EGFR, ALK and ROS1, followed by optional testing of 5 recommended genes was the cheapest approach, at an average cost of 2227 USD (95% CI, 1733–2794 USD). Upfront NGS, on the other hand, was 2500 USD [12]. Since then, however, the number of recommended molecular analyses has increased, adding cost, delays in diagnosis and complexity to this algorithm. NGS compares favourably to single-gene assays in terms of sensitivity [13, 14]. For instance, among light smokers (under 15 pack-years) with lung adenocarcinoma, without oncogenic alterations in 11 single-gene assays, 65% of patients were diagnosed with targetable drivers with NGS analyses [13]. Among patients with EGFR mutations, NGS can detect insertions and deletions or single nucleotide variations that would be overlooked with polymerase chain reaction (PCR) assays [14]. While the clinical significance of some alterations can be variable, identifying them can grant access to clinical trials or impact treatment decisions. NGS can identify deleterious mutations in tumour suppressor genes rather than just gain of function mutations, as with PCR [8]. Similarly, NGS can assess copy number variations and predict amplifications. Detection of rearrangements requires special techniques (hybrid capture) for NGS performed on DNA, and are best identified by RNA sequencing. Taking all of these matters into account, while the sequence of immunohistochemistry, polymerase chain reaction, fluorescence in situ hybridization and Sanger sequencing were commonly used nearly a decade ago, today, upfront NGS has become the gold standard, when and where it is available and would allow targeted therapies [15].

Despite recommendations and the proven paramount importance of molecular testing in NSCLC, far too often, these tests are not performed, even in developed countries. A recent International Association for the Study of Lung Cancer (IASLC) survey concluded that over 60% of responders believed molecular testing was performed in fewer than 50% of patients in their country [16]. These global results mirrored those of developed countries such as the United States and Canada [17]. Finally, when physicians requested molecular testing, they commonly asked for EGFR (99%), ALK (95%) and ROS1 (79%), while other alterations were requested under 50% of the time.

With the widespread availability of NGS but the continued concern of insufficient tissue, there is an ever-growing interest in liquid biopsies and circulating tumour DNA (ctDNA) based NGS analyses. CtDNA can identify discordant alterations compared to tissue biopsies, including subclonal drivers of resistance to therapy. Its use could offer access to further targeted therapies even in case of negative tissue analyses [18]. While plasma NGS can identify targets, its sensitivity is lower than that of tissue NGS. Positivity on plasma NGS has an over 96% concordance with tissue findings, while tissue positivity is associated with only approximately 60% detection in plasma [19]. This matches the 60–65% sensitivity of plasma NGS for EGFR mutations in subgroups analyses from the LUX LUNG 3 and LUX LUNG 6 trials of afatinib [20]. Interestingly, the same analysis showed a 28% sensitivity for serum NGS, establishing that plasma NGS is a more reliable option [21]. This is also the recommendation of the IALSC [8]. Across a number of prospective and retrospective analyses in patients with advanced oncogene-driven lung cancer, there is a 68–80% concordance between tissue and plasma NGS, with a sensitivity of 58–85% and a specificity of 87–100% [8].

Plasma NGS have the advantage of better reflecting tumor heterogeneity, particularly when searching for mechanisms of resistance to targeted therapies, and can also shorten the time to identification of an oncogenic driver [22]. As such, plasma NGS represents hope for detection of alterations that could tailor therapy and prolong a patient’s life, yet it has many limitations. First, it is important to stress that not all tumours will shed enough DNA into the bloodstream for NGS to reliably detect its presence. This will inherently limit sensitivity. A low disease burden and indolent growth may be more likely to yield false-negative plasma NGS results. Next, it is imperative that the baseline analysis be conducted on a sample before any therapy. Even one or two weeks of treatment can greatly diminish the variant allele frequency and create false-negative plasma results [23].

Today, plasma and tissue biopsies are complementary. As diagnostic techniques evolve, the practicality of liquid biopsies will likely lead to their ubiquitous use.

### Targetable oncogenic drivers in NSCLC

**Fig. 2:**
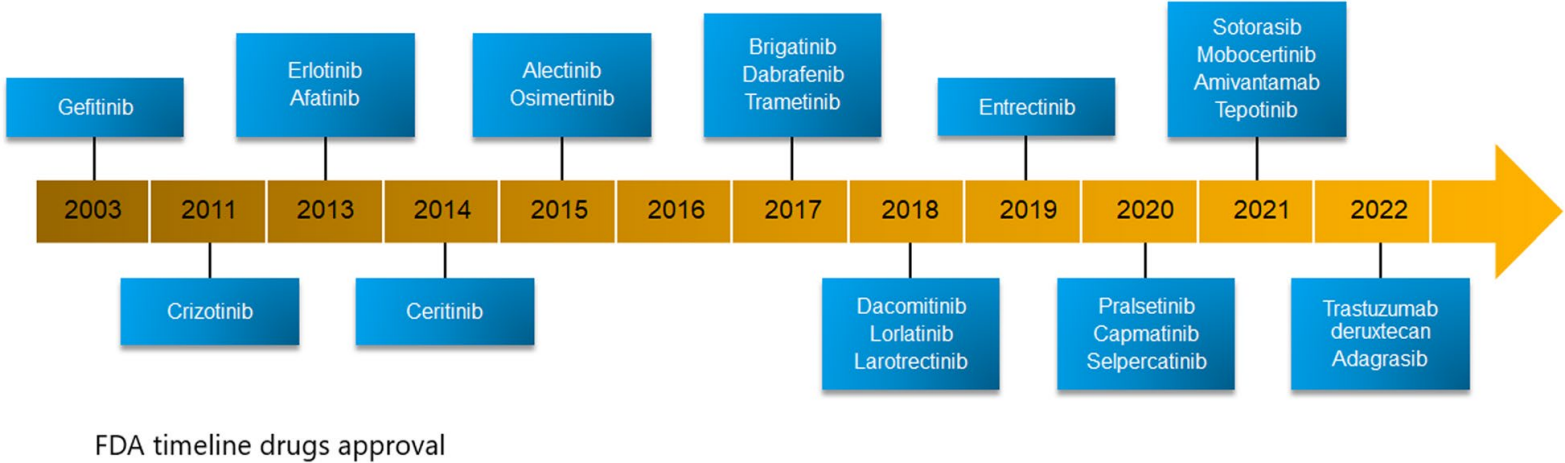
FDA timeline of drug approvals. Since 2003, there has been a rapid acceleration of drug development and approvals for molecular targeted therapies in NSCLC. Today, both kinase inhibitors and antibody–drug conjugates are approved

**Fig. 3:**
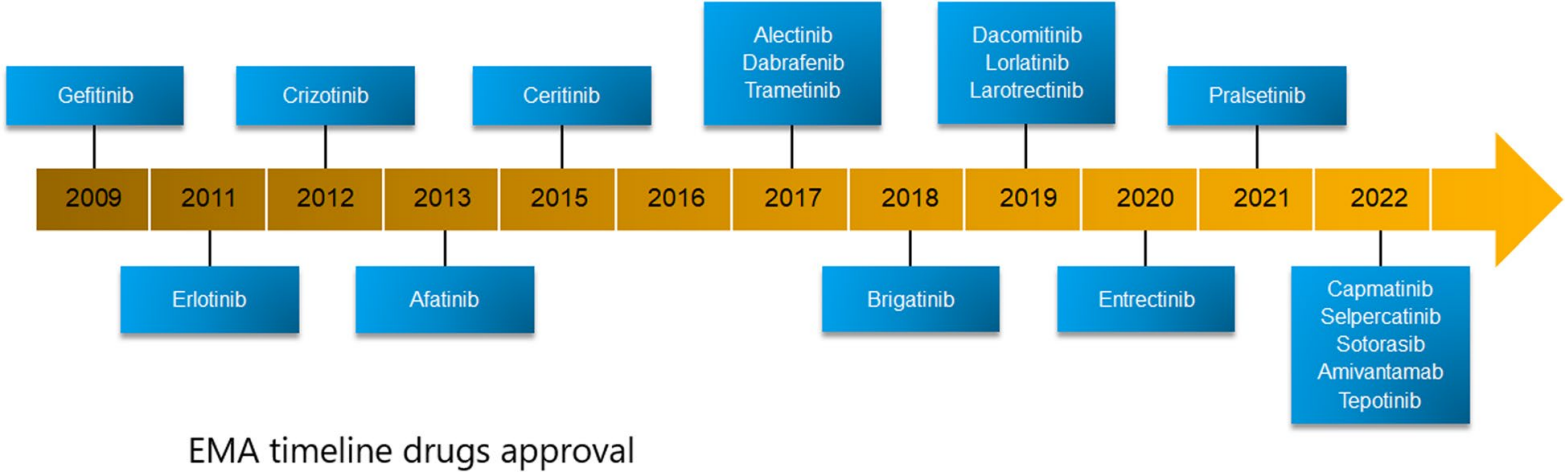
EMA timeline of drug approvals. Since 2003, there has been a rapid acceleration of drug development and approvals for molecular targeted therapies in NSCLC. Today, both kinase inhibitors and antibody–drug conjugates are approved

We will discuss oncogenic targets in advanced NSCLC and their current therapies stating whether there have been approved by local health body authorities such as Food and DRUG Administration (FDA) and European Medicine Agency (EMA) (Figs. 2 and 3), as well as those on the horizon.

### EGFR

The erythroblastic leukemia oncogene B (*ErbB*) receptor tyrosine kinase pathway has an important role in proliferation, tumorigenesis and apoptosis. It is also known as the human epidermal growth factor receptor (*HER*). This receptor family comprises four cell-surface receptors: epidermal growth factor receptor (*EGFR* or *HER1*), *HER2*, *HER3* and *HER4*.

On a physiological level, each HER receptor is a monomer in its inactive state. When bound to its ligand, it activates signalling by dimerizing with another HER-family receptor. The downstream signalling cascade stimulates cell proliferation [24]. Constitutional activation or dysregulation of HER receptors is a common oncogenic driver. EGFR is a cell surface protein that binds epidermal growth factor, its ligand, inducing dimerization, phosphorylation of tyrosine residues and downstream signalling activation of the RAS-MAP kinase pathways, PI3K-AKT-mTOR and JAK-STAT [25]. While EGFR is expressed in normal tissues, particularly in the skin and gastrointestinal tract, it was identified as being over-expressed in various cancers, particularly in lung cancer [26]. It should be noted that over-expression from amplification does not necessarily have the same clinical impact as gain of function mutations.

In NSCLC, the most common HER mutation is in *EGFR*. It was identified in lung cancer in 2004 and was the first target of genome-oriented therapy to be approved by the FDA. In the untreated setting, EGFR mutation is mutually exclusive with most other oncogenic alterations. After selective therapeutic pressure, however, it can co-exist with novel alterations that appear as genomic-resistance bypass mechanisms.

In lung adenocarcinoma, the prevalence of EGFR mutations varies widely by geographic distribution. In Asian countries, and among Asian populations, EGFR mutations can be detected in more than 50% of patients with lung adenocarcinoma. In Western populations, its prevalence hovers around 15% [27]. In lung adenocarcinoma, EGFR mutations are more common among Asian, young, female, non or light smokers [28]. EGFR mutations encompass what are referred to as classical mutations, including exon 19 deletions or exon 21 L858R point mutations, accounting for 90% of EGFR alterations, as well as uncommon mutations. The latter comprise a large number of mutations, the most common being EGFR exon 20 insertions, which represent up to 2.5% of all lung adenocarcinomas and 6% of *EGFR*-mutant NSCLC cases [29].

### Common EGFR mutations

At the turn of the millennium, erlotinib and gefitinib, two first-generation EGFR kinase inhibitors, appeared to have very modest in vivo activity. Both responses rates, varying between 10 and 20% and progression-free survival, at around 3 months, were very disappointing in an unselected, previously treated, advanced NSCLC population [30, 31].

Interestingly, non-smokers and Asian patients appeared to derive more benefit from these treatments. The reason for these findings would come to light with the discovery of an enriched incidence of somatic EGFR mutations in these populations [32]. On a biological level, the majority of EGFR mutations detected were exon 19 deletions and exon 21 L858R point mutations. These exhibited 100 times greater sensitivity to first generation TKI EGFR inhibition than wild-type EGFR [33]. It is important to have higher selectivity for EGFR mutants than wild-type EGFR to avoid off-target toxicity, in this case, cutaneous side effects. These discoveries heralded the era of genome-oriented targeted therapy trials in NSCLC. Early phase then randomized trials confirmed the greater efficacy of EGFR inhibition than chemotherapy among patients with EGFR mutant advanced NSCLC. In contrast to results seen in an unselected population, these trials found high response rates, rising to roughly 75%, and a median progression-free survival of around 10 months [34, 35]. Thanks to a higher clinical efficacy and lower toxicity of TKIs compared to chemotherapy, TKIs were established as standard front-line therapy for EGFR mutant advanced NSCLC. This is a key discovery and highlights the importance of patient selection and personalized medicine.

Over time, all patients treated with first generation EGFR inhibitors develop an acquired resistance within the first year of treatment. One additional EGFR mutation appears to account for 50–60% of resistance mechanisms, namely the exon 20 T790M gatekeeper mutation in the drug-binding site of the ATP-pocket [36, 37]. Novel drugs were developed in order to overcome this resistance. In vitro, the second-generation irreversible pan-HER inhibitor, afatinib, showed improved activity against T790M. However, clinically, it appeared to fare no better than first generation TKIs and induced T790M resistance mechanisms at a similar frequency [36]. Furthermore, the use of afatinib after failure of first-generation TKIs did not improve survival nor show significant responses in the LUX-Lung 1 trial [38]. This drug and dacomitinib both produced positive front-line trials against chemotherapy and gefitinib, respectively, but their use is limited by gastrointestinal and skin toxicity and the development of the third-generation EGFR TKI, osimertinib [39, 40]. Subsequently, the phase IIb LUX-Lung 7 trial did not show a survival difference between afatinib and gefitinib in the front-line setting [41].

The third-generation osimertinib was designed to inhibit common EGFR mutations and particularly the T790M resistance mutation [42]. Osimertinib has a low affinity to certain efflux transporters like permeability glycoprotein, leading to longer lasting central nervous system activity than previous generation TKIs [43]. The central nervous system activity became a major advantage, as brain metastases develop in over a quarter of patients with EGFR mutant advanced NSCLC [44]. Osimertinib first proved its superiority over platinum-pemetrexed chemotherapy among patients with confirmed T790M acquired resistance to previous generation EGFR inhibitors, with a response rate of 71% compared to 31% with chemotherapy and more than doubled progression-free survival of 10.1 versus 4.4 months, respectively. Similarly, grade 3 or higher toxicity was less likely in the Osimertinib arm, at 23 vs 47% [45].

In the phase III FLAURA trial, Osimertinib was compared to first-generation EGFR TKIs in the front-line setting. It demonstrated improved progression-free survival, with a median of 18.9 versus 10.2 months, with an hazard ratio (HR) 0.46and improved overall survival at 38.6 versus 31.8 months (HR 0.80), respectively, and better central nervous system penetration, with metastases appearing in 6% versus 15% of patients [46, 47]. The progression-free survival and favourable toxicity profile established osimertinib as a front-line standard of care, and the overall survival update cemented the role of osimertinib as the preferred front-line therapy.

Major efforts are currently underway to improve the results obtained with osimertinib in first-line setting. No other third generation TKIs have demonstrated superiority over osimertinib to-date, given its long-term efficacy and safety profile. Recent data from the phase 3 LASER301 trial shows that the third generation EGFR TKI, Lazertinib, which also has anti T790M activity, is superior to gefitinib, with a median progression-free survival of 20.6 months versus 9.7 months and a 76% response rate [48], while data on OS were not reported (NR). These results mirror those of recent Chinese phase 3 trials, including the AENEAS trial comparing aumolertinib to gefitinib [49], the FURLONG trial, comparing furmonertinib [50] to gefitinib, and befotertinib [51]. Current frontline trials are using osimertinib as a control arm. Some are based on the improved PFS data when adding chemotherapy to front-line gefitinib [52, 53]. For instance, osimertinib monotherapy is being studied in combination with chemotherapy in the FLAURA2 trial (NCT04035486). A similar trial is comparing osimertinib to another third generation TKI, aumolertinib, with or without chemotherapy in the setting in the TREBLE study (NCT05493501). Osimertinib alone is being compared to the addition of anti-VEGF bevacizumab (NCT04181060), though the similar phase 2 BOOSTER trial in second-line with T790M mutations did not meet its primary endpoint of progression free survival. The ongoing phase 3 MARIPOSA trial is comparing osimertinib to the bispecific antibody amivantamab and lazertinib [54]. Table 1 summarizes currently published front-line phase 3 EGFR trials (Table 1).

**Table 1:** Selected first-line phase 3 trials in EGFR mutant NSCLC

Trial	Clinical trials ID	Treatments	Objective response rate (%)	Median progression-free survival (months)	Median overall survival (months)
FLAURA	NCT02296125	Osimertinib vs gefitinib or erlotinib	80 vs 76	18.9 vs 10.2 (HR 0.46)	38.6 vs 31.8 (HR 0.80)
ARCHER	NCT01774721	Dacomitinib vs gefitinib	75 vs 72	14.7 vs 9.2 (HR 0.59)	34.1 vs 26.8 (HR 0.76)
LUX-Lung 7	NCT01466660	Afatinib vs gefitinib	70 vs 56	11 vs 10.9 (HR 0.73)	27.9 vs 24.05 (HR 0.86)
LASER301	NCT04248829	Lazertinib vs gefitinib	76 vs 76	20.6 vs 9.7 (HR 0.45)	NR vs NR (HR 0.74)
FURLONG	NCT03787992	Furmonertinib vs gefitinib	89 vs 84	20.8 vs 11.1 (HR 0.44)	NR vs NR (HR 0.94)
AENEAS	NCT03849768	Aumolertinib vs gefitinib	74 vs 72	19.3 vs 9.9 (HR 0.46)	NR vs NR
Lu et al	NCT04206072	Befotertinib vs icotinib	76 vs 78	22.1 vs 13.8 (HR 0.49)	NR vs NR

Upon progression on osimertinib, the COMPEL trial is assessing the impact of continuing osimertinib with subsequent chemotherapy to the use of chemotherapy alone (NCT04765059). The utility of immunotherapy post osimertinib remains unclear. In an exploratory subgroup analysis of IMpower150, atezolizumab, bevacizumab and a platinum doublet appear to show a survival improvement over chemotherapy alone [55]. Next, Keynote 789 failed to demonstrate a benefit with the addition of pembrolizumab to chemotherapy after EGFR TKI failure [56]. Similarly, Checkmate 722 failed to show improved survival outcomes with the addition of nivolumab to chemotherapy after first or second-line osimertinib [57]. However, the recent ORIENT-31 trial found a statistically significant progression-free survival benefit for the anti-PD1 antibody, sintilimab, with a bevacizumab biosimilar (PFS 7.2 months) and a trend towards a benefit without it(PFS 5.5 months), compared to chemotherapy alone (PFS 4.3 months) [58].

A mechanism of resistance to osimertinib is detected in 52% of biopsied patients with disease progression. The most common escape mechanisms are MET amplifications at about 18%, and small cell transformation and EGFR amplifications, at 14% each [59]. Other bypass mechanisms have been described, such as the emergence of KRAS or BRAF mutations, or fusions. A recent case series showed *RET* fusions as resistance mechanisms to Osimertinib, with a 50% ORR and 83% DCR with the addition of selpercatinib [60]. A quarter of patients develop on-target resistance mechanisms, especially C797X mutations.

Currently, fourth generation TKIs are being developed with the aim of overcoming on-target resistance to osimertinib. Some allosteric EGFR inhibitors, that is, those which do not compete for a binding site but cause a conformational change instead, have reported results. EAI045 binds EGFR adjacent to the C-helix, which does not target the ATP-pocket but regulates EGFR enzymatic activity, while EGFR is in an inactive conformation. It is very potent against classical L858R mutations and T790M mutations in vitro. It also appears active against the resistant C797S mutations in combination with cetuximab in mouse models. However, in vivo, this molecule has not shown promising activity as a monotherapy, and a combination therapy is known to add significant unwanted wild-type EGFR toxicity [61, 62]. Another allosteric inhibitor, JBJ-04–125-02, has demonstrated anti L858R, T790M and C797S activity in vitro and in vivo.[63] There has also been a focus on central-nervous system penetration due to the tropism of EGFR mutant NSCLC for the brain. Furthermore, new models are highly selective to spare EGFR wild-type, in order to decrease toxicity. BLU-945 was designed to have potent central-nervous system activity and can target classical exon 19 or 21 mutations, as well as T790M and C797S. It has shown robust in vitro and in vivo activity in Osimertinib refractory tumours [64, 65]. BLU-701 is similar but fails to target T790M. It will be assessed alone and as combination therapy. Both are still in early phase trials (NCT04862780 and NCT05153408).

Another promising approach is that of antibody–drug conjugates. The phase 3 MARIPOSA-2 trial is comparing a platinum-doublet arm to chemotherapy with amivantamab and a third arm with the addition of lazertinib in the post-osimertinib setting (NCT04988295). Patritumab-deruxtecan targets HER3, a member of the EGFR family ubiquitously expressed in EGFR mutant NSCLC. After the success of the phase 2 HERTHENA-Lung 01 trial which demonstrated a 39% ORR (95% CI 26–52) and 8.2 month median PFS (95% CI 4.4–8.3) in the post-TKI setting (86% prior Osimertinib), this drug received FDA breakthrough therapy designation in December 2021 [66]. The HERTHENA-Lung 02 phase 3 trial is ongoing, comparing patritumab-deruxtecan to chemotherapy after osimertinib (NCT05338970). Finally, the TROP2-directed datopotamab-deruxtecan demonstrated activity in a number of oncogene-addicted NSCLC subtypes in the phase 1 TROPION-PanTumor01 trial. The ORR was 35% (95% CI 19.7–53.5) and the median DOR was 9.5 months (95% CI 3.3-not reached) among 29 patients with EGFR mutations, 3 with ALK rearrangements and one with each RET and ROS1 rearrangements [67].

### Uncommon EGFR mutations

The widespread adoption of NGS in NSCLC allowed the detection of many previously unknown variants of EGFR. Uncommon EGFR mutations comprise a large number of alterations and represent roughly 10% of all EGFR mutations in NSCLC. The most common are EGFR exon 20 insertions, which represent 2.5% of all lung adenocarcinomas and 6% of *EGFR*-mutations in NSCLC [29, 68]. In the French real-world database, these mutations were found in a similar population to common EGFR mutations, with regards to age and smoking status [69].

It is important to note that exon 20 insertions comprise over 100 distinct variants in NSCLC, demonstrating their heterogeneity [70]. The p.V769_D770insASV variant is more common among people over 65 years old, while p.A763_Y764insFQEA and p.H773_V774insNPH variants are more prevalent in those under 65 years of age. Other variants, such as p.V769_D770insASV, p.V774_C775insHV, p.H773_V774insNPH and p.D770_N771insSVD tend to be diagnosed in women and non-smokers [71]. Exon 20 insertions generally are poorly responsive to first-generation EGFR therapy, while second and third-generation TKIs demonstrate only limited in vivo efficacy [72]. Exon 20 insertions cause steric hindrance, blocking the binding pocket of classical EGFR TKIs. One exception is the A763_Y7764insFQEA variant, which is sensitive to all EGFR TKIs [73]. Exon 20 insertions are associated with a worse prognosis compared to classical EGFR mutations in advanced NSCLC [74].

After years of development, there are now two FDA approved therapies for EGFR exon 20 insertions and many other drugs in ongoing trials. Mobocertinib is an EGFR and HER2 targeting TKI with selective inhibition of exon 20 insertions over wild-type EGFR [75]. The phase I-II trial among 28 patients found a 43% response rate and 7.3 month progression-free survival. The phase 2 EXCLAIM expansion cohort found similar survival results among 114 platinum pre-treated patients. Despite the high selectivity against wild-type EGFR of this TKI, there are typical EGFR inhibitor toxicities including diarrhea and nausea in 92 and 28% of patients, respectively. Skin rashes appeared among 45% of patients. These results led to the ongoing phase III EXLCAIM-2 trial, comparing mobocertinib to platinum-double chemotherapy in the front-line setting (NCT04129502) and to FDA approval in April 2020. Other TKIs targeting EGFR exon 20 insertions are under development as zipalertinib, sunvozertinib or furmonertinib, with the aim of a better efficacy-toxicity balance.

Amivantamab, the other approved therapy against EGFR exon 20 insertion mutations, is a bispecific antibody targeting EGFR and MET. Binding both receptors may cause a synergistic inhibition of downstream signalling pathways. Amivantamab downregulates EGFR and MET in cell-lines with EGFR exon 20 insertions and in classical canonical EGFR mutant cell lines [76]. In the CHRYSALIS study, 81 patients were treated with amivantamab with a response rate of 40% and progression-free survival of 8.3 months [77]. Skin rash was common, reported in 86% of patients. However, only 4% had grade 3 rashes. Another significant adverse event was the risk of infusion-related reactions, with an incidence of 66% but again only 3% grade 3. One study compared it to poziotinib, another exon 20 insertion TKI being developed, and found far less skin toxicity with amivantamab [78]. Amivantamab was the first FDA approved EGFR exon 20 insertion therapy, with breakthrough therapy designation awarded in March 2020 and full FDA approval in May 2021. Recently, the PAPILLON study which is a randomized trial of amivantamab plus chemotherapy versus platinum-based chemotherapy alone in the front-line setting was found to significantly improve the PFS [79] (Table 2).

**Table 2:** First-line phase 3 trials in EGFR exon 20 insertion NSCLC

Trial	Clinical trials ID	Treatments	Primary endpoint
PAPILLON	NCT04538664	Amivantamab with chemotherapy vs chemotherapy alone	PFS: endpoint met
EXCLAIM-2	NCT04129502	Mobocertinib vs chemotherapy	PFS: endpoint not met

Other mutations include G719X, L861Q and S768I. A post hoc analysis from the LUX-Lung 2, 3, and 6 trials found a response rate of 71% and progression-free survival of 10.7 months among patients harbouring these uncommon EGFR mutations and treated with afatinib [80]. In the UNICORN study, a real-world analysis of osimertinib in uncommon EGFR mutations, the most common were G719X and L861Q, detected in 30% and 20% of patients respectively. Interestingly, these mutations were more common in women, Caucasians and at a median age of 64. The results of this real-world trial mirrored the Korean phase II KCSG-LU15-09 study, with a response rate around 47% and progression-free survival of 8.8 months [68, 81].

#### HER2

The human epidermal growth factor 2 (HER2) gene is located on the chromosome 17q12. It encodes HER2, a transmembrane protein, composed of 3 main components: the extracellular ligand binding domain, the trans-membrane α-helical segment and the intracellular tyrosine kinase domain. Ligand binding induces receptor dimerization, and auto-phosphorylation and activation of the intracellular kinase domain [82]. HER2 is unique among the HER family of receptors because it is able to be activated through homodimerization or heterodimerization with other HER proteins in a ligand-independent manner [29]. There is no known natural ligand of HER2. HER2 activation triggers major downstream proliferation and anti-apoptotic signaling pathways, including MAPK, STAT and PI3K/AKT/mTOR [83].

*HER2* can induce oncogenesis through gene amplification, activating mutations and over-expression. These alterations have a varying degree of sensitivity to *HER2* targeting therapy. To date, the greatest efficacy in targeting HER2 has been achieved in breast and gastro-oesophageal tumour subtypes [84, 85].

The functional implications of a HER2 mutation depend on the mutation site. In NSCLC, most HER2 mutations are exon 20 insertions and occur in the kinase domain. These represent up to 3% of NSCLC, primarily among patients who are younger, non-smokers and with an adenocarcinoma [83].

Meanwhile, *HER2* amplifications are rarely de novo alterations in NSCLC but are among the most frequent mechanisms of acquired resistance, especially to EGFR-targeting therapy. They occur in roughly 2% of NSCLC upfront but up to 13% after therapy [37].

Recently the efficacy and safety data from 48 patients with treatment-naive, advanced *HER2*-mutant NSCLC treated with the pan-HER receptor tyrosine kinase inhibitor pyrotinib were published [86]. This is a phase II study adaptive umbrella trial consisting of a criteria-fulfilled (CF) cohort and a compassionate use (CU) cohort under expanded eligibility criteria, and a prospective real-world study (RWS). In the CF cohort (*n* = 28), the primary endpoint was reached with an objective response rate of 35.7% after pyrotinib treatment. Secondary endpoints included disease control rate (89.3%), mPFS 7.3 months, median OS 14.3 months and toxicity, which was acceptable, with grade 3 or 4 treatment-related adverse events occurring in three patients (10.7%). In the CU cohort (*n* = 12) the ORR was rate 16.7%, with DCR of 83.4%, median PFS of 4.7 months and median OS of 14.2 months after pyrotinib treatment. The RWS cohort (*n* = 8) had no responses to physician’s therapy of choice, while median PFS and OS were 3.0 and 12.2 months, respectively.

European cohort retrospective data on trastuzumab based regimens in 57 patients with pre-treated HER2 mutant NSCLC indicated limited activity, with a 50% response-rate but only 5 months of median progression-free survival [87]. A prospective phase 2 trial of the antibody–drug conjugate ado-trastuzumab emtansine in a similar patient population mirrored these results, with a 44% response rate and 5 month progression-free survival [88]. Both of these datasets had manageable toxicity profiles. A more recent antibody–drug conjugate, trastuzumab-deruxtecan, has shown what appears to be the greatest activity to date in HER2 mutant advanced NSCLC. The phase 2 DESTINY-Lung01 trial included two cohorts of patients with previously treated advanced NSCLC: HER2 amplifications and HER2 mutations. The preliminary results from 91 patients in the HER2 mutation cohort revealed a 55% response rate, 8.2 month median progression-free survival and 17.8 month overall survival [89]. However, toxicity remains challenging, with 46% grade 3 or higher drug-related adverse events, including 19% neutropenia. Drug-related interstitial lung disease was diagnosed in 26% of patients and caused two toxic deaths.

The DESTINY-Lung02 trial, a randomized phase 2 study, recently compared the 6.4mg/kg 3-weekly regimen used in DESTINY-Lung01 to a 5.4mg/kg regimen. The results favoured the lower dose, with numerically higher response-rates of 53.8% compared to 42.9% in the higher dose cohort, but more importantly, nearly half the grade 3 or higher toxicity, drug discontinuation and dose reductions in the lower dose cohort [90]. The ongoing phase 3 DESTINY-Lung04 trial is comparing trastuzumab-deruxtecan to platinum-doublet chemotherapy (NCT05048797). Trastuzumab-deruxtecan currently holds an FDA accelerated approval status [91].

Many EGFR exon 20 insertion targeted therapies have a broad HER spectrum and are being evaluated in HER2 mutations as well. An example is poziotinib, an oral HER2 inhibitor, active against exon 20 insertions. In the Zenith20 trial, a multi-cohort, open-label phase 2 study, among 90 heavily pretreated patients, there was an ORR of 27.8% (95% CI 18.9–38.2) and 70% DCR. This translated to a median PFS of 5.5 months (95% CI 3.9–5.8), regardless of the HER2 mutation subtype. However, grade 3 or higher toxicity was a limiting factor, including 49% rash, 26% diarrhea and 24% stomatitis [92]. Given the adverse event profile and poor efficacy, poziotinib was not approved by the FDA (Table 3).

**Table 3:** Selected phase 1/2 trials for HER2 mutant NSCLC

Trial	Clinical trials ID	Treatments	Objective response rate (%)	Median duration of response (months)	Median overall survival (months)
Zenith20-1	NCT03318939	Poziotinib	27.8	5.5	NR
Destiny-Lung02	NCT04644237	Trastuzumab-deruxtecan 5.4mg/m2 vs 6.4mg/m2	53.8 vs 42.9	Not estimable vs 5.9	NR
Liu et al	NCT03605602	Pyrotinib	35.7	7.3	14.3

#### KRAS

*Rat sarcoma *(*RAS*) genes (*KRAS*, *NRAS*, *HRAS)* are one of the most frequently mutated oncogenes in human malignancies. They are identified in 1 in 7 cancer cases. Furthermore, KRAS is ubiquitous, expressed as a membrane bound protein in all human cells. In NSCLC, KRAS mutations are the most commonly detected alterations, found in approximately 30% of cancers [93].

KRAS proteins are members of the guanosine triphosphate (GTP)ase family, with a major role in intracellular signalling. When confronted to extracellular signals, these GTPases control switching between the active GTP-bound and inactive GDP-bound states. The transition between GTP and GDP is regulated by GTPase activating proteins (GAPs) and guanine nucleotide exchange factors (GEFs) [94–96] via the release of GDP and the binding of GTP. Many GEFs have been identified, including Son of Sevenless (SOS1) which plays a significant role in KRAS activation and is being studied as a potential therapeutic target [97].

The active GTP bound RAS initiates major signalling cascades that control division, proliferation, differentiation, and survival. These include the RAS-RAF-MEK-ERK mitogen-activated protein kinase (MAPK) pathway, involved in cell-cycle regulation and, ultimately, controlling proliferation. Another major pathway regulated by RAS is PI3K-AKT-mTOR signalling, essential in maintaining cell survival. Finally, KRAS activation leads to RAS-dependent tumour growth via the RAL pathway and vesicle trafficking via the tumour invasion and metastasis-inducing protein 1 (TIAM1-RAC1) pathway [98, 99].

Many KRAS mutations exist, and their functional impact varies tremendously due to their diverging biology. For instance, KRAS substitutions in G12, G13 and Q61 inhibit GTP hydrolysis, thus preventing the switch to an inactive KRAS state. This results in constitutive KRAS activation and downstream signalling. On the other hand, KRAS A146T, which is the most common KRAS mutation in gastrointestinal cancers, does not involve dysregulated GTP hydrolysis. Instead, it increases nucleotides exchanges, leading to increased KRAS-GTP formation, ultimately resulting in a less potent oncogenic signal [100].

Distinct *KRAS* mutations can involve different downstream signalling cascades. For instance, cell lines of mutant KRAS-G12D demonstrated preferential activation of the PI3K–AKT pathway [101]. Meanwhile, in vitro studies of KRAS G12C and KRAS G12V revealed enhanced RAS-related protein (RAL) A/B signalling but reduced levels of phosphorylated AKT compared to wild-type KRAS signalling or other KRAS mutations [102].

In NSCLC, the prevalence of KRAS mutations in Western populations can reach approximately 30%, while these are lower in Asian populations. They can be detected by next-generation sequencing, which has revealed that the most common alterations are substitution mutations in codon 12 (90%) or 13 (6%) and 61 (1%) [103]. By far the most common KRAS alteration in NSCLC is the G12C mutation. It is detected in roughly 13% of NSCLC and represents 41% of all KRAS mutations in NSCLC [104]. KRAS G12V represents approximately 21% of KRAS alterations in NSCLC and G12D, 17% [105, 106]. KRAS mutations in lung cancer are found predominantly in adenocarcinoma, representing 37% of cases, while roughly 4% of squamous cell carcinomas harbour these alterations. They appear more common among Caucasians than Asians, at 26% and 11% respectively, slightly more common among females than males, at 31 versus 24% and three times more common among smokers than non-smokers, with a 30% and 11% prevalence, respectively [106, 107]. This distribution, particularly regarding ethnicity and smoking status, makes KRAS mutations stand out from most other oncogenic drivers. Interestingly, there are differences between KRAS subtypes, with G12C being more prevalent among patients with significant smoking history, while G12D alterations are most common among light or non-smokers [93].

KRAS was long considered an undruggable target in part due to the very high affinity of GTP for KRAS. The last few years have brought significant change with two currently approved KRAS G12C inhibitors. These inhibitors bind to the cysteine residue at G12C and covalently block the KRAS protein in its GDP-bound ‘inactive’ state [96].

The phase I/II Codebreak100 trial included 59 previously treated patients with advanced NSCLC harbouring KRAS G12C mutations. Patients received sotorasib, a small molecule that blocks the KRAS G12C protein in its inactive position, with an objective response rate of 32% and a median duration of response of nearly 11 months [108]. CodeBreaK200 is the first phase III randomized trial of a KRAS inhibitor, wherein sotorasib (*n* = 171) was compared against docetaxel (n = 174) in second-line treatment of metastatic NSCLC. At a median follow-up of 17.7 months, the study met its primary endpoint of improving PFS, with median PFS of 5.6 months versus 4.5 months in sotorasib and docetaxel arms, respectively (HR 0.66, 95% CI 0.51–0.86; p = 0.0017). The 12-month progression-free survival rates favoured the sotorasib arm, at 24.8%, compared to 10.1% with docetaxel. The response rate was 28.1% versus 13.2% and disease control rates 82.5% versus 60.3% in the sotorasib and docetaxel arms, respectively [109, 110]. The most common grade 3 adverse events were gastrointestinal, including diarrhea, nausea and liver enzyme elevation. Disspointingly, there was no difference in median overall survival (10.6 months for sotorasib versus 11.3 months for docetaxel), though it should be noted that 34% of patients in the control arm crossed-over to sotorasib. While the results are not as impressive as expected, this represents the first phase 3 trial confirming the efficacy of a KRAS G12C inhibitor (Table 4).

**Table 4:** Selected trials for KRAS G12C mutant NSCLC

Trial	Clinical trials ID	Phase	Treatments	Objective response rate (%)	Median progression-free survival (months)	Median overall survival (months)
CodeBreak 100	NCT03600883	1/2	Sotorasib	32	6.3	12.5
Krystal-1	NCT03785249	1/2	Adagrasib	42.9	6.5	12.6
CodeBreak 200	NCT04303780	3	Sotorasib vs docetaxel	28.1 vs 13.2	5.6 vs 4.5 (HR 0.66)	10.6 vs 11.3

*Abbreviations*: *NSCLC* non-small cell lung cancer, *KRAS* Kirsten rat sarcoma, *HR* hazard ratio

Adagrasib is the second KRAS G12C inhibitor to be approved. KRYSTAL-1 is a phase I/II trial that included 116 previously treated patients with advanced KRAS G12C mutated NSCLC [111]. The efficacy of adagrasib was evaluable in 112 patients, with a response rate of 42.9% [112]. Median PFS was 6.5 months and OS, 12.3 months. The toxicity profile appeared similar to that of sotorasib, though it had fewer gastrointestinal and hepatic adverse events. The phase 3 KRYSTAL-12 trial, comparing adagrasib to docetaxel in the second-line setting, will read out soon (NCT04685135).

With more clinical experience and trial evidence, subtle differences between sotorasib and adagrasib are gaining attention. Liver toxicity seems to preclude combination of sotorasib with anti-PD(L)-1, which is not the case in preliminary data for adagrasib. For instance, adagrasib led to an intracranial ORR and DCR of 31.6% and 84.2%, respectively, in patients with previously untreated intracranial metastasis [113]. On the other hand, among patients with treated, stable brain metastasis in the CodeBreaK200 trial, sotorasib induced a numerically longer time to CNS recurrence than docetaxel but the difference was not statistically significant (9.6 months vs 5.4 months (HR 0.84, 95% CI: 0.32–2.19, *p* = 0.37) [114]. Other KRAS G12C-GDP complex inhibitors that have shown promising activity are JDQ443, GDC-6036, JAB-21822,

Current results show lower efficacy than with other oncogenic drivers, with response rates under 50%. Some hypotheses may explain the intrinsic resistance to these small molecule inhibitors. The first is that KRAS mutant tumour may not be exclusively RAS-driven. For instance, RAS-independent activation of PI3K/AKT/mTOR downstream pathways may induce resistance to KRAS targeting [115]. An alternative intrinsic resistance mechanism could be the intratumour heterogeneous distribution of KRAS mutations within the same cancer, leading to the presence of non-G12C clones [116]. A lower efficacy may also be seen as these studies were in patients with previously treated NSCLC, increasing the possibility of other acquired signaling pathway alterations in addition to KRAS G12C. In the phase II SCARLET trial of sotorasib in combination with carboplatin and pemetrexed in previously untreated non-squamous NSCLC, the ORR was 88.9%, however, median PFS was disappointing at 5.7 months [117].

In addition to primary resistance, both on-target and off-target mechanisms of adaptive resistance have been identified. A frequently observed on-target resistance mechanism to sotorasib is the emergence of a secondary KRAS G13D mutation, found in 23% of resistant clones. On the other hand, adagrasib induces KRAS Q99L resistance mutations in almost 53% of cases. Both can induce KRAS Y96D and A59S and a number of other rarer resistance point mutations [118]. These involve conformational changes in the switch pocket II, impairing the ability of small molecule inhibitors to bind KRAS. Identifying resistance mechanisms is important for drug development, to allow novel G12C inhibitors to cover common escape pathways. Preclinical data for the novel KRAS inhibitor RM-018 show efficacy against acquired Y96D mutations [119]. Sotorasib and adagrasib differ in resistance mechanisms but until novel drugs target these, the clinical impact of these differences remains unknown.

Off-target resistance mechanisms include epithelial-to-mesenchymal transformation, bypass signalling pathways and cell senescence. Epithelial-to-mesenchymal transformation in tumours resistant to sotorasib was associated with a downregulation of E-cadherin and upregulation of vimentin. For adagrasib, it was associated with the transformation of adenocarcinoma into squamous cell carcinoma [120]. Sotorasib resistance has also been linked to FGFR and IGFR1 activation in the presence of epithelial to mesenchymal transformation [121].

The most common off-target bypass signalling, detected in sotorasib and adagrasib resistant tumours, includes *MET* amplification, BRAF, NRAS, MAP2K1 mutations and RET, ALK, BRAF, FGFR and RAF1 fusions [120]. KRAS inhibition can also amplify upstream drivers including receptor tyrosine kinases/ Src homology 2 domain-containing phosphatase 2 (RTKs/SHP2). G12C inhibitors suppress ERK-mediated inhibition of RTKs/SHP2, activating the above-mentioned pathways. Ultimately, this restores MAPK signaling [122].

Transition to senescence may be another acquired resistance mechanism. Aurora kinase A allows KRAS G12C to escape from a quiescent, drug-induced G0 state, meaning that enhanced signalling could explain a transition to senescence [123]. The SPARK trial, assessing ctDNA identified molecular resistance mechanisms, may shed further light on the biology of resistance (NCT05272423).

Novel KRAS inhibitors such as ‘active’ state inhibitors, allosteric inhibitors, and KRAS degraders are in earlier phases of development. Combination approaches are also being tested, with immune checkpoint inhibitors, SHP2 and SOS1 inhibitors, among others [124–126]. G12C inhibition has expanded the horizon to develop inhibitors to other KRAS mutations. For instance, MRTX1133, a KRAS G12D inhibitor entered in phase 1 clinical trial (NCT05737706). More recently, a non-covalent pan-KRAS inhibitor was developed, which showed preclinical inhibition of multiple KRAS mutations in GDP-bound state [127]. The field of KRAS ‘drugging’ is in its nascency and in the coming years, we expect to see a complete shift in paradigm for treatment of RAS-driven malignancies.

#### ALK

The fusion of the anaplastic lymphoma kinase gene, *ALK,* to the nucleolar protein gene, NPM1 was identified in 1994 in specific lymphoma subtypes [128]. Over a decade later, the echinoderm microtubule-associated protein-like 4 (EML4)-ALK rearrangement was discovered in NSCLC [129]. Today, more than 90 distinct fusion partners for ALK have been identified. *ALK* rearrangements are found in a similar population to EGFR mutations, namely among patients with lung adenocarcinoma, light or never-smokers, and a younger age at diagnosis [130]. They are found in roughly 4% of patients with NSCLC [131].

*ALK* is thought to be involved in development of the nervous system during fetal development but its expression is suppressed post-natally. Therefore, ALK, when post-natally expressed, is almost always aberrant and disease related, barring in rare neuronal or endothelial cells. *ALK* rearrangements with a promoter gene such as EML4 leads to expression of fusion ALK proteins. These ALK proteins constitutively dimerize and induce ALK kinase activation, leading to uncontrolled downstream signalling in the RAS-MAPK, P3K–AKT-mTOR and JAK–STAT pathways. This results in tumour proliferation and cell survival [132].

In addition to rearrangements, oncogenic ALK amplifications can also be seen. They also induce constitutive activation via the hyperphosphorylation of the SHcC docking protein, located near the substrate of the ALK receptor [133]. ALK amplification has been described in a number of cancers including NSCLC, though its optimal treatment is unclear.

Point mutations in *ALK* mainly develop in response to *ALK* TKIs as acquired on-target resistance mechanisms [134]. The secondary point mutations, C1156Y and L1196M, were the first demonstrated to confer drug resistance to TKIs. Since, many others have been identified, as resistance to first and second generation ALK TKIs, including the G1202R and I1171X mutations [135].

When discussing the treatment, we refer exclusively to ALK rearranged NSCLC. The first targeted therapy for ALK rearranged advanced lung cancer was the multikinase inhibitor, crizotinib. A phase I trial showed a 60% response rate and 9.7 month progression-free survival [136]. This led to FDA approval for crizotinib in 2011. Subsequently, randomized phase 3 trials found crizotinib to be superior to standard platinum-based chemotherapy in the front-line and to docetaxel or pemetrexed in second-line setting, with a more tolerable safety profile as well [137, 138]. In the front-line setting, for example, crizotinib had a 74% response rate, compared to 45% for chemotherapy and median progression-free survival rates were 10.9 versus 7 months, respectively. Common side effects of crizotinib included vision impairment, upper and lower gastrointestinal symptoms and peripheral oedema.

Patients invariably develop resistance to crizotinib, largely due to acquiring on-target resistance mutations in the ALK domain. This led to the development of second-generation ALK TKIs, including ceritinib, brigatinib and alectinib. These second-generation ALK TKIs were first assessed in crizotinib resistant patients and were successful in overcoming many of the common resistance mechanisms [139]. Furthermore, they exhibited improved CNS activity owing to better CNS penetration.

Second-generation ALK TKIs were subsequently compared to crizotinib as front-line therapy, demonstrating superior outcomes in the phase 3 ALEX, ALTA-1 and eXalt-3 trials for alectinib, brigatinib and ensartinib, respectively [140–143], changing the paradigm of front-line management. These second-generation treatments had improved response rates at 71–82.9% compared to 60–75% with crizotinib, progression-free survival rates, 24–34.8 months versus 10.9–12.7 months and intracranial response rates, at 63.8–78% compared to 21.1–29% for crizotinib. Furthermore, they generally had a more favourable toxicity profile. Overall survival updates for second-generation ALK TKIs have shown impressive results, including 62.5% of patients alive at 5 years in the ALEX trial [143].

The third-generation ALK and ROS1 TKI, lorlatinib, was developed to overcome on-target resistance to previous generation ALK TKIs, particularly the G1202R mutation. Another aim was to improve CNS efficacy, due to the high prevalence of CNS invasion in ALK rearranged NSCLC. This was achieved by improving drug concentration in the CNS by decreasing drug efflux from the CNS in Pgp-overexpressing cells [144, 145]. In a phase 2 trial among ALK TKI pre-treated patients, lorlatinib had a response rate of 47%, and an intracranial response rate of 63%. In the treatment naïve cohort, the response rate was 90% [145]. It should be noted, however, that the toxicity profile of lorlatinib differs from other ALK TKIs. Lorlatinib is also associated with gastrointestinal adverse events and oedema but has two major additional adverse event categories: hyperlipidemia and neurocognitive side effects. Lorlatinib was approved by the FDA in 2018 for previously treated patients with NSCLC harbouring ALK rearrangements. A subsequent study found lorlatinib to be more effective among patients in whom secondary ALK mutations were detected than in those without identified resistance mechanisms. This can be explained by a continued ALK-dependence in the former population [146].

The recent CROWN trial compared front-line lorlatinib to crizotinib. It demonstrated an improved response rate of 76% versus 58% and intracranial response rate of 82% versus 23% [146]. The three-year update shows a progression-free advantage, with median rates of 36.7 compared to 29.3 months, for lorlatinib and crizotinib, respectively [147]. The impressive CNS efficacy was maintained. Toxicity, however, was higher in the lorlatinib, with 76% of grade 3–4 adverse events, versus 57% in the crizotinib arm. Lorlatinib was approved by the FDA in the front-line setting in March 2021, though the optimal treatment sequence remains a contentious topic, given the tolerance profile. Front-line trials are summarized in Table 5.

**Table 5:** Selected first-line phase 3 trials in ALK rearranged NSCLC

Trial	Clinical trials ID	Treatments	Objective response rate (%)	Median progression-free survival (months)	Median overall survival (months)
ALEX	NCT02075840	Alectinib vs crizotinib	82.9 vs 75.5	34.8 vs 10.9 (HR 0.43)	NR vs NR (HR 0.76)
ALTA-1L	NCT02737501	Brigatinib vs crizotinib	74 vs 62	24.0 vs 11.1 (HR 0.48)	NR vs NR (HR 0.81)
CROWN	NCT03052608	Lorlatinib vs crizotinib	77 vs 59	NR vs 9.3 (HR 0.28)	NR vs NR

After second generation ALK inhibitors, secondary ALK point mutations are the most common resistance mechanism. With alectinib, for example, G1202R appears in 30% of patients and I1171X in a further 15%) [148]. Resistance to lorlatinib, on the other hand, includes only approximately 25% of on-target mutations [149]. Furthermore, these mutations are generally compound point-mutations, such as G1202R/L1196M, I1171N/D1203N or C1156Y/L1198F. This can be explained by the potency wide spectrum of activity of lorlatinib. Novel TKIs aiming to overcome these compound mutations are being tested. For example, TPX-0131 is a compact TKI designed to fit inside ALK’s ATP-binding pocket, regardless of secondary mutations. In murine model, it has shown efficacy against different ALK point mutations, including compound ones [150]. It is currently under investigation in a phase 1 trial (NCT04849273). Another novel ALK TKI is NVL-655, a highly CNS penetrant TKI with activity against some of the common compound mutations seen in lorlatinib resistant tumours [151].

Should these molecules overcome the resistance to lorlatinib, they are also likely to induce further escape mechanisms, whether on or off-target. Drug combinations will certainly need to be assessed.

## ROS1

*ROS1*-rearrangements were first detected as *FIG-ROS1* gene fusions in glioblastoma. Since then, ROS1 rearrangements have been detected in multiple tumour types, including cholangiocarcinoma (8.7%), ovarian cancer (0.5%), and lung cancer. In NSCLC, the EZR–ROS1 fusion was the first ROS1 rearrangement. Today, at least 23 distinct fusion variants have been discovered in NSCLC, the most common being CD74-ROS1, which is found in approximately half of cases [152]. ROS1 is a true oncogenic driver and is usually mutually exclusive with other primary driver alterations [153]. It is detected in 1–2% of NSCLC [154]. ROS1 shares the same patient demographics as ALK: it is more prevalent in younger patients, with a median age of 49, females, non-smokers, Asians, and is predominantly found in adenocarcinoma [155, 156]. Clinically, ROS1 diseases have up to 5 times more risk of thromboembolic events than other NSCLC, though this does not impact survival [157].

Rearrangement involves the fusion of a segment of ROS1 that encompasses the entire tyrosine kinase domain with one its partner proteins. ROS1 encodes a tyrosine kinase receptor which belongs to the family of insulin receptors and has a similar structure to ALK proteins. No natural ligand has been identified. To date, there is no clear answer about divergent roles or clinical correlates of different fusion partners. There are conflicting data about CD74-ROS1 fusion partners and a higher risk of brain metastases. Brain metastases are frequent in ROS1-positive NSCLC, with an incidence of up to 35%. However, the correlation with CD74-ROS1 may simply reflect that this is the most common type of ROS1 rearrangement [88, 158], rather than a more aggressive phenotype. While ROS1 fusion kinases have various mechanisms of action, they all are constitutively activated and promote oncogenic downstream signalling pathways, including ESYT1 [154]. ROS1 kinase activation triggers major signalling cascades including the RAS–MAPK, PI3K-AKT-mTOR and JAK-STAT3 pathways, inducing proliferation and cell survival.

It is worthwhile to note that other ROS1 alterations exist in cancer, including overexpression, amplification and splice variants that lead to truncated ROS1 protein that lacks an intracellular domain. Unlike fusions, the pathogenicity of these alterations is unclear.

Early trials of ROS1 in NSCLC were subgroups of ALK TKI studies. In the phase I PROFILE 1001 trial, 50 patients with advanced NSCLC harbouring ROS1 rearrangements were treated with crizotinib. Among the patients, of whom over 80% were previously treated with chemotherapy, there was a 72% response rate and 19.2 month progression-free survival [159]. The median overall survival was 51 months, and this preceded the advent of certain subsequent TKI options [139]. Crizotinib consistently demonstrated response rates in the 70% range in phase 2 trials of patients with advanced NSCLC harbouring ROS1 rearrangements, though CNS progression was a major problem [160], as it represented the first and sole site of progression in 47% of the patients[158, 161]. The toxicity profile is similar to that reported in ALK trials, with mainly gastrointestinal adverse events and peripheral oedema.

Treatment failure with crizotinib is due to two main reasons: brain progression and on-target secondary mutations, the latter in up to 60% of patients [162]. Some infrequent causes of resistance comprise bypass signalling pathways including EGFR, KIT and KRAS, phenotypic changes like EMT and transformation to small cell lung cancer [163, 164]. When looking at the on-target acquired resistance to crizotinib, seven point mutations have been identified to date: G2032R, G2032K, D2033N, S1986Y, S1986F, L1951R and L2026M. The solvent front mutation G2032R, which is structurally analogous to G1202R mutation in ALK, stands out as it represents a staggering 41% of these mutations [163, 164]. In light of the above, novel therapies for ROS1 needed to focus on CNS penetration and point mutations, particularly G2032R.

In a phase 2 Korean study, 30 patients with ROS1 positive, crizotinib-naïve advanced NSCLC were treated with the second-generation ALK and ROS1 TKI, ceritinib. The outcomes appear similar to those seen with crizotinib, with a response rate of 67%, median progression-free survival of 19.3 months and overall survival of 24 months [165]. There were 37% grade 3 or greater adverse events and gastrointestinal toxicity was a limiting factor for treatment. The study also included two patients who had received previous crizotinib. Neither responded to ceritinib.

Entrectinib is a TKI with activity against NTRK and ROS1. In a phase 1–2 trial of 53 patients with advanced, ROS1 rearranged NSCLC, the response rate reached 77%, with a median progression-free survival of 19 months and duration of response of 24.6 months. The intracranial response rate was 55%, with a median duration of response of nearly 13 months in patients with CNS disease [18]. The patient population included 32% who were treatment-naïve and 38% with baseline CNS metastases. The toxicity profile included grade 3–4 adverse events in 34% of patients, the most common being weight gain and neutropenia. Only 5% of patients discontinued treatment. The FDA approved entrectinib for ROS1-positive NSCLC in August 2019.

Lorlatinib was assessed in phase 1–2 study of 69 patients with advanced NSCLC harbouring ROS1 rearrangements [146]. Among 40 patients who had progressed on crizotinib, the response rate was 35% and the median progression-free survival was 8.5 months. Among 21 crizotinib-naive patients, lorlatinib exhibited an improved response-rate of 62%, with a median progression-free survival of 19.3 months. While these results appear to mirror those of front-line crizotinib, the main appeal of lorlatinib in the treatment-naïve setting is its intracranial activity, with intracranial response rates of 64% in this cohort and 50% post crizotinib. Among 8 patients who received a TKI other than crizotinib prior to lorlatinib, the response rate and progression-free survival were 13% and 5.6 months, respectively. Grade 3–4 adverse events were reported in 49% of patients, including hypertriglyceridemia, hypercholesterolemia and neurotoxicity.

The PFROST real world dataset showed a similar efficacy of lorlatinib in crizotinib pretreated patients, with a response rate of 39%. Unfortunately, patients with G2032R acquired resistance mutations to crizotinib failed to derive benefit from lorlatinib. A French expanded access programme, LORLATU, and an Asian retrospective trial showed similar results [166–168].

Repotrectinib is a next generation TKI that targets ROS1, ALK and NTRK. It has a 90-fold higher potency for ROS1 than crizotinib, targets G2032R, and has improved blood–brain-barrier penetration [159, 169]. In the phase 1–2 TRIDENT-1 trial, repotrectinib was administered to different cohorts of patients with advanced ROS1 rearranged NSCLC. Among 71 TKI-naïve patients, the response rate was 78.9%, and at the 18 month follow-up, progression-free survival and duration of response were still immature. In the cohort that received 1 prior TKI, the response rate was 37.5%. Among those who received two prior TKIs, of whom all received crizotinib, 71% lorlatinib and 17% entrectinib, the response rate dropped to 28%. The 12-month progression-free survival in these 3 cohorts was 80%, 44% and 7%, respectively [170]. Of particular interest, the response rate among patients with *ROS1* G2032R was 59%. Most adverse events were grade 1–2, including dizziness, dysgueusia, constipation, paresthesia, anemia, nausea and fatigue. The front-line efficacy of these different agents among treatment naïve patients are summarized (Table 6).

**Table 6:** Selected first-line results from phase 1/2 trials in ROS1 rearranged NSCLC

Trial	Clinical trials ID	Treatments	Objective response rate (%)	Median progression-free survival (months)	Median overall survival (months)
PROFILE-1001	NCT00585195	Crizotinib	72	19.3	NR
TRIDENT-1	NCT03093116	Repotrectinib	78.9	NR	NR
Shaw et al	NCT01970865	Lorlatinib	62	19.3	NR
Pooled analysis of STARTRK-1, STARTRK-2, ALKA-372	NA	Entrectinib in treatment-naïve patients	68.7	17.7	47.7

Other novel agents such as the G2032R targeting ROS1-NTRK TKI, taletrectinib (DS-6051b), are currently in early phase trials. Preclinical data suggest high potency against the acquired G2032R/D2033N solvent front mutations, though similar response rates to other TKIs have been reported [171]. In a phase I trial among crizotinib pretreated patients, Taletrectinib had a 33.3% response rate [172].

The major challenge in ROS1 remains acquired resistance mechanisms. On lorlatinib, a third of patients develop on-target mutations and a further 10% develop MET amplifications [173]. Further data are required about combining MET inhibitors with ROS1 TKIs, as has been done in EGFR mutant NSCLC with MET amplified acquired resistance. Entrectinib can induce KRAS G12C as a resistance mechanism, for which combinations should also be explored. More data is needed on mutations like ROS1 L2086F, which cause resistance to all currently approved ROS1 inhibitors. Finally, it remains unclear whether sequencing with crizotinib or giving a novel inhibitor upfront is the optimal management similarly to ALK rearrangements [174]. Today, we choose based on the presence of brain metastases and toxicity profiles.

### BRAF

The MAPK pathway is among the main pathways transducing extracellular signalling into cellular responses. BRAF, an intracellular protein kinase, plays a critical role downstream of RAS in these pathways, sending signals from membrane receptors to cell nuclei [175]. This oncogene, located on chromosome 7, is important in cell growth, proliferation, differentiation and apoptosis.

There are around 200 identified BRAF mutations across cancer types, and these occur in approximately 5.5% of all cancer in humans. In solid tumours, their incidence is highest in melanoma and papillary thyroid cancer, in which they are detected in about 50% of cases, in colorectal cancer, at 10% and in lung adenocarcinoma, at 2–8% [176, 177]. A BRAF mutation induces structural changes with constitutive activation of the MAPK signalling cascade.

The V600E activating mutation is the most common variant across tumour types, representing 90% of BRAF mutations, though it accounts for merely 50% of BRAF mutations in lung cancer. The BRAF V600E subtype is more common among women, and in aggressive micropapillary histological subtypes. It can be found among smokers and non-smokers [177]. Meanwhile, non-V600E variants are commonly diagnosed in males and those with a smoking history. It is worth noting that V600E mutations are true oncogenic drivers and are mutually exclusive with other druggable oncogenes in the treatment-naïve setting, while non-V600E can coexist with other oncogenes including KRAS mutations [178]. The clinical relevance of each variant is difficult to ascertain, though what is of particular importance is the sensitivity of distinct BRAF mutations to targeted therapy.

Platinum based chemotherapy appears to underperform in patients with advanced NSCLC harbouring BRAF mutations [179]. When it comes to PD-1 inhibitors, in spite of a tendency of high PD-L1 expression in BRAF mutant NSCLC, there is no correlation between PD-L1 expression and drug efficacy [107]. Patients with NSCLC harbouring *BRAF* mutations have a limited response to immunotherapy [2, 180]. Patients with non-V600E appear to derive more benefit from checkpoint inhibitors than those with V600E, but the latter have higher overall survival, likely due to targeted therapy options.

Dabrafenib and vemurafenib, novel-generation *BRAF* inhibitors, are ATP-competitive inhibitors of *BRAF* kinase. Vemurafenib is effective in targeting *BRAF*-V600 mutants in NSCLC but ineffective in other variants [181]. Dabrafenib also showed efficacy in V600E variants in a phase 2 trial [182].

To overcome resistance via activation of downstream MAPK pathways, dabrafenib was combined with trametinib, a MEK inhibitor. This combination was tested in phase 2 trials in the first and second line. Front-line, the response rate was 64%, progression-free survival 14.6 months, and overall survival 24.6 months [90]. There was a similar response rate in second-line [182]. Today, front-line combination approaches are recommended for V600E advanced NSCLC. A major advantage of BRAF V600E inhibitors over chemoimmunotherapy is their intracranial activity. The toxicity profile of dual BRAF-MEK inhibition can be challenging at times, with pyrexia being the most common toxicity, seen in 56% of patients, often requiring dose reduction or interruption. Other notable adverse events include cardiomyopathy, dermatologic toxicities, ocular toxicity such as retinal detachment and retinal vein occlusion, hypertension, hyperglycemia and secondary skin cancer [183]. Recently, results from the phase II PHAROS trial, which studied the combination of BRAF inhibitor, encorafenib and MEK inhibitor, binimetinib, were reported [184]. The study met its primary endpoint of ORR, and 75% of treatment-naïve patients had an objective response. In previously treated patients, the ORR was 46%. PFS and OS data were not mature at the time of study publication (Table 7). Most importantly, the rates of pyrexia were much lower with this regimen and the most frequently reported TRAEs were nausea, diarrhea, vomiting, and fatigue.

**Table 7:** Selected first-line results from phase 2 trials in BRAF mutant NSCLC

Trial	Clinical trials ID	Treatments	Objective response rate (%)	Median progression-free survival (months)	Median overall survival (months)
Planchard et al	NCT01336634	Dabrafenib/trametinib	64	14.6	24.6
PHAROS	NCT03915951	Encorafenib/ binimetinib	75	NR	NR

Despite activity, acquired resistance mechanisms emerge. It appears that MAPK bypass activation is the most common cause of acquired resistance, via other RAF isoforms including CRAF and ARAF, for example [185]. KRAS G12D and G12D have also been described as resistance mechanisms [186], as has PTEN inactivation [187].

Some resistance mechanisms may be overcome by novel multi-RAF and downstream ERK1/2 inhibitors, like LXH254 and LTT462, which are in early phase trials for BRAF or KRAS mutant NSCLC [188]. VS-6766 is one such RAF/MEK clamp that is being studied in BRAF mutant NSCLC (RAMP 202 NCT).

### RET

The Rearranged during transfection (RET) gene is located on chromosome 10 (10q11.2) and is translated into a transmembranous proto-oncogene receptor tyrosine kinase. Interestingly, it has an intracellular kinase domain which is 37% homologous with that of ALK [189] and shares some signalling pathways. Neurotrophic ligand-induced RET activation leads to dimerization and autophosphorylation of the RET kinase domains, thus activating downstream transduction pathways including RAS/MAPK, PI3K/AKT/mTOR and JAK/STAT cascades [189]. In physiologic conditions, RET plays a role in the enteric nervous system and the development of the urogenital tract.

Germline alterations of *RET* are involved in various diseases. Loss of function is linked to decreased RET receptors in developing gut tissues, impeding neuroblast migration and enteric nervous system maturation, as described in Hirschsprung’s disease. In contrast, activating mutations are linked to multiple endocrine neoplasia type 2A [190, 191], comprising medullary thyroid cancer, parathyroid adenoma and pheochromocytoma.

Non-germline *RET* dysregulations in cancer can stem from *RET* gene rearrangements which lead to the production of a chimeric RET fusion protein, and to constitutive activation of *RET* promoting cell proliferation and survival [192]. RET rearrangements occur in 1–2% of NSCLC. The two most common fusion partners are the kinesin family 5B (KIF5B), identified in 70–90% of RET-positive NSCLC, and the coiled coil domain containing-6 (CCDC6), in 10–25% [193]. There are many less common fusion partners including *NCOA4, ZNF477P, ERCC1, HTR4*, *TRIM33* and *CLIP1* [194]*.* RET rearranged NSCLC are enriched among patients who are younger, non-smokers, and those with adenocarcinoma [189]. They have an aggressive disease course, with a high risk of brain metastases [195]. RET rearrangements are true oncogenic drivers and are mutually exclusive with *EGFR, KRAS, BRAF* mutations and *ALK* and ROS1 rearrangements [196].

*RET* fusions can be diagnosed with different techniques but immunohistochemistry for RET can have weak staining patterns, limiting its efficacy, and RT-PCR interrogates a limited number of gene partners and would overlook novel fusions [194]. NGS is the preferred approach, particularly RNA sequencing, which identifies known or unknown fusion partners and quantifies fusion transcripts [197].

Until recently, target therapy for NSCLC with RET rearrangements consisted of broad spectrum multikinase inhibitors. Given the rarity of this alteration, a global registry collected data of patients with RET-rearranged NSCLCs revealing that cabozantinib, sunitinib and vandetanib were of limited efficacy, with response rates of 37%, 22% and 18%, respectively [198]. The highly selective RET inhibitors that followed displayed improved efficacy and lower toxicity.

Selpercatinib, a highly selective oral, CNS penetrant RET inhibitor, was assessed in the phase I/II LIBRETTO 001 trial in patients with advanced NSCLC with RET rearrangements [199]. Among 105 chemotherapy pretreated patients, the response rate was 64%, while it reached 85% among 39 treatment naive patients. The median progression-free survival was 16.5 months. The intracranial response rate was 91%. The safety profile was tolerable, with the most common grade 3–4 adverse events including hypertension, increased liver enzymes, hyponatremia and lymphopenia. Selpercatinib was granted FDA accelerated approval and a phase III front-line trial, LIBRETTO 431, is ongoing, comparing it to a platinum-doublet ± pembrolizumab, with a positive PFS readout in the interim analysis [200].

Pralsetinib is another highly selective *RET* TKI, with activity against many *RET* fusions and potent CNS activity [148]. In the phase I/II ARROW trial, 121 patients with *RET*-rearranged NSCLC were included [148]. The distribution of fusion partners in this trial mirrored known evidence, with *KIF5B* in 66% and *CCDC6* in 13% of patients. Ninety-two patients had received prior platinum-doublet chemotherapy, with a 61% response rate. Among treatment naïve patients, there was a 70% response rate. The median progression-free survival was 17.1 months. Grade 3 or higher adverse events mainly consisted of neutropenia, hypertension and anaemia. Currently available data are summarized in the table below (Table 8).

**Table 8:** Selected first-line results from phase 1/2 trials in RET rearranged NSCLC

Trial	Clinical trials ID	Treatments	Objective response rate (%)	Median progression-free survival (months)	Median overall survival (months)
Libretto-001	NCT03157128	Selpercatinib	85	16.5	NR
ARROW	NCT03037385	Praseltinib	70	17.1	NR

Praseltinib was granted accelerated approval by the FDA in September 2020. Meanwhile, AcceleRET is an ongoing phase 3 trial, comparing praseltinib to platinum-based chemotherapy ± pembrolizumab in RET-rearranged advanced NSCLC (NCT04222972).

Resistance to multikinase inhibitors has revealed *RET* V804M gatekeeper and *RET* S904F mutations [201]. These acquired resistance mutations are sensitive to selective RET TKIs [202]. Data are limited regarding resistance to novel RET TKIs. The emergence of *RET* G810 (R, S, or C) solvent front mutations has been linked to resistance to selpercatinib in the context of multiple RET fusion partners [203]. Novel therapies such as LOX-18228 are striving to overcome this mechanism. Off-target resistance has been linked to MET and KRAS amplifications and NTRK fusions, warranting the study of combination approaches [204]. In a case series combining selpercatinib with crizotinib in light of an acquired MET-amplification, responses lasted up to 10 months [205].

#### MET

*MET* is a proto-oncogene *MET* on chromosome 7q21-q31, which encodes a transmembrane receptor tyrosine kinase known as MET or hepatocyte growth factor receptor. When MET binds its ligand, it dimerizes, autophosphorylates and induces intracellular catalytic activity of its tyrosine kinase domain [206]. This leads to downstream RAS/MAPK, PI3K/AKT/mTOR, FAK, STAT, RAC/PAK and Wnt/β-catenin signalling cascades. MET dysregulation induces cell proliferation, migration, invasion, survival, angiogenesis and histologic transition from epithelial to mesenchymal [207]. Aberrant MET activation can result from heterogenous alterations, comprising amplifications or copy number gains, rare gene fusions, exon 14 skipping, protein overexpression and activating point mutations in MET’s kinase domain. All potentially lead to constitutional MET receptor activation, with downstream proliferation signaling [208–210]. Exon 14 skipping is the most important MET-related oncogenic driver in the treatment-naïve setting in advanced NSCLC. It involves aberrant splicing and skipping of exon 14 in the messenger RNA transcript and is the result of either missense mutations, insertions and/or deletions. Exon 14 contains the binding site for CBL, an E3 ubiquitin ligase, which when absent, leads to impaired MET ubiquitination [211]. This leads to MET ‘immortalization’ and aberrant MET downstream signalling.

MET exon 14 skipping mutations have an incidence of 3–5% in NSCLC. They are predominantly in adenocarcinoma and are enriched in the sarcomatoid histology, by up to 15% [123]. *MET* amplifications are also mainly in adenocarcinoma, where their de novo incidence is 1–5% [208]. MET exon 14 skipping and amplifications both are over-represented among non-smokers, yet smokers are more common than in patients with EGFR or ALK-driven NSCLC, with 61–74% and 77% of patients being smokers among patients with exon 14 skipping and amplification, respectively [212]. Exon 14 skipping is rarely found with coexisting oncogenic alterations, except for *MET* amplifications, while *MET* amplifications, especially at low levels, can co-occur with other oncogenic drivers [213]. As such, amplifications should only be considered oncogenic drivers at high amplification or gene copy levels [214]. It should be noted that *MET* amplifications have a particular relevance as acquired resistance mechanisms under selective pressure of targeted therapies in many oncogene-driven NSCLC.

Diagnosis of MET alterations is complex. Amplification is usually defined as a MET to centromere P7 (CEP7) ratio greater than 2. There should be more than 5 signals per cell for copy number gain. A MET to CEP7 ratio greater than 5 is predictive of a true MET-driven tumour but represents only 0.34% of lung adenocarcinoma [214]. RT-PCR can assess copy numbers but cannot distinguish between amplification and polysomy. However, gene copy numbers above 10 appear predictive of MET-driven diseases [208]. Immunohistochemistry is unreliable for MET as it only detects protein overexpression and is poorly correlated with amplification [215, 216]; however, it can be used for selecting patients candidates to antibody–drug conjugates targeting the MET protein.

MET exon 14 skipping is far more common and requires a diagnostic approach that can detect mutations between exon 13 and 15. RT-PCR can be employed, with a high sensitivity (100%) and specificity (97.4%) compared to DNA NGS. DNA sequencing detects genomic variants that alter splicing sites. In contrast, RNA NGS detects fusions between exon 13 and 15, which are the result of all exon 14 skipping mechanisms, making it the preferred diagnostic approach [217].

The last decade has seen many MET inhibitors, including multikinase inhibitors like crizotinib, cabozantinib and more recently, selective MET inhibitors such as capmatinib, tepotinib, tivantinib and monoclonal antibodies with limited efficacy, onartuzumab, emibetuzumab, ficlatuzumab and rilotumumab.

Immunotherapy appears to have limited efficacy in MET-driven NSCLC, with a 16% response rate and roughly 3 month median progression-free survival [218]. In contrast, MET TKIs are an effective treatment option, at least for exon 14 skipping. They offer a promising treatment option in patients with exon 14 skipping. In these patients, the phase 1–2 PROFILE 1001 demonstrated a 32% response rate, 7.3 month progression-free survival and 20.5 month overall survival among patients treated with crizotinib [219].

More recently, the phase 2 VISON trial assessed the highly selective MET-inhibitor, tepotinib, in patients with advanced NSCLC with MET exon 14 skipping [220]. The response rate was 46% and median progression-free survival of 8.5 months. It should be noted that whether the diagnosis was on tissue or a liquid biopsy, results were similar. Capmatinib was also assessed in the phase 2 GEOMETRY-mono-1 trial, for patients with exon 14 skipping, with a 68% response rate and 9.7 month progression-free survival in treatment-naïve patients. Among previously treated patients, the response rate was 41% and progression-free survival, 5.5 months [215] (Table 9). In patients with MET amplifications defined by a gene copy number of 10 or higher, the response rate was 40% in previously untreated patients, and 29% in previously treated patients. In both trials, common side effects were gastrointestinal toxicity and peripheral oedema, a side effect that is often difficult to manage, which are a class effect with MET inhibitors.

**Table 9:** Selected first-line results from phase 2 trials in MET exon 14 skipping NSCLC

Trial	Clinical trials ID	Treatments	Objective response rate (%)	Median progression-free survival (months)	Median overall survival (months)
PROFILE 1001	NCT00585195	Crizotinib	25	NR	NR
VISION	NCT02864992	Tepotinib	44.9	8.5	NR
Geometry-Mono-1	NCT02414139	Capmatinib	68	9.7	NR

In the phase 1 CHRYSALIS trial evaluating the activity of amivantamab in previously treated patients with NSCLC harbouring EGFR exon 20 insertions, one patient had a MET amplification and achieved a partial response [221]. Amivantamab was assessed in a cohort of 55 patients with MET exon 14 skipping in the CHRYSALIS trial, with an ORR of 33% overall but 57% in previously untreated patients. Further research is warranted about the role of this bispecific antibody in MET alterations [222]. Telisotuzumab vedotin, a MET ADC is also currently being studied in a similar setting, after progression on prior EGFR TKI and acquiring MET amplification.

Acquired resistance develops to MET inhibitors. Secondary MET mutations in residues D1228 and Y1230 appear for selective TKIs. In contrast, crizotinib induces the solvent front G1163R mutation, which is sensitive to other MET inhibitors. In vitro, acquired resistance mutations to novel selective TKIs remain sensitive to multikinase inhibitors, while resistance to multikinase inhibitors, including mutations at L1195 and F1200, is sensitive to selective MET inhibitors. Therefore, depending on the on-target resistance mechanism, changing classes of MET inhibitors may be an option. However, other off-target mechanisms including KRAS and PIK3 mutations can develop and drug combinations require further study [223, 224].

Finally, MET amplification is a common mechanism of resistance to other oncogenic drivers, especially EGFR. The INSIGHT-2 trial demonstrated the efficacy and safety of adding tepotinib to osimertinib among patients with MET-amplification as a resistance mechanism to first-line osimertinib in advanced EGFR mutant NSCLC. Approximately half the patients responded to the combination [225]. The TATTON trial demonstrated a 33% ORR with the addition of savolitinib in this setting [226], leading to the ongoing randomized phase 3 SAFFRON trial comparing this combination to chemotherapy. Further combinations are being evaluated, including capmatinib with osimertinib (NCT04816214).

#### NTRK

The *neurotrophic receptor tyrosine kinase* (*NTRK1, NTRK2, NTRK3*) genes encode tropomyosin receptor kinases (TRKA, TRKB and TRKC). The ligands of NTRK are neutrophic factors, involved in the development, survival and proliferation of nerve cells [227].

When *NTRK* undergoes a translocation with a fusion partner including *ETV6*, *LMNA* and *TPM3*, the neurotrophic factors it encodes are translated into NTRK fusion proteins that lead to constitutive activation of tyrosine kinases. More than 25 fusion partners have been identified [228, 229], but all result in a overexpression of constitutively activated TRK kinase, which trigger downstream signalling pathways such as MAPK and PI3K/AKT/mTOR leading to uncontrolled cell proliferation [229–231].

*NTRK* fusions are very infrequent across most solid tumours, representing 0.5% of all cancers [232]. They are more common in a number of rare cancer types, including secretory breast carcinoma, infantile fibrosarcoma, infantile non-brain-stem glioblastoma and mesoblastic nephromas, in which they can be found in up to 90% of patients [228, 233]. In more common cancer types, NTRK fusions are very rare, for example, they account for 0.2% to 3.3% of NSCLC [229].

The diagnosis of *NTRK* fusions can be made through pan-TRK IHC. It is inexpensive, quick, has a high sensitivity but a low specificity [234]. Given the latter, a confirmation test should be used before basing therapy on IHC results. A RT-PCR test is rapid and can be used to confirm the diagnosis but is suboptimal for screening, given that it could overlook unknown fusion partners. FISH can also be used as a confirmatory test but requires more material, as a separate assay must be used for each *NTRK* gene. Furthermore, while FISH is highly sensitive in detecting fusions with canonical breakpoints, it can result in false negatives when breakpoints involve noncanonical sites. There FISH can have limited sensitivity for *NTRK* [235]. Today, the gold standard for screening and confirmation of NTRK fusions in diseases in which these alterations are rare, such as lung cancer, is upfront RNA-based NGS, as part of a broader molecular analysis. Properly diagnosing this rare entity is important given the therapeutic advances for *NTRK*.

The ROS1 and ALK inhibitor, entrectinib, also targets TRKA, TRKB and TRKC. It was designed to have high CNS penetration. In the phase 1–2 trial in patients with advanced cancers with NTRK fusions, entrectinib exhibited a 57% response rate among 54 patients, and a 70% response rate among the 10 patients with NSCLC. The median progression-free survival in the entire cohort was 11.2 months, and in the NSCLC subgroup, 14.9 months [236]. Among patients with brain metastases, there was a 54.5% intracranial response rate [237]. Dysgeusia, fatigue and constipation were the most frequent adverse events, while anemia and weight gain topped the grade 3–4 list, at 5% each and neurocognitive effects were rare serious adverse events.

Larotrectinib is an oral highly selective pan-TRK inhibitor designed to have low CNS penetration to avoid neurocognitive toxicity. In a pooled analysis of phase I/II trials among 159 children and adults with advanced tumours harbouring NTRK fusions, the response rate was 79%. Among the 12 patients with NSCLC, there was a 75% response rate. The median progression-free survival was 28.3 months and overall survival, 44.4 months. The most common adverse events were fatigue, liver enzyme increases and cough, while the 13% of grade 3–4 side effects included liver enzyme increases and haematological toxicity [237].

Both of these agents were granted accelerated FDA approval in advanced NTRK-positive tumours (Table 10). As with other highly effective targeted therapies, acquired resistance is inevitable with NTRK inhibitors. A number of resistance mechanisms have been identified, leading to the development of second-generation inhibitors. Selirectinib and repotrectinib are designed to overcome solvent-front mutations and xDFG substitutions [238].

**Table 10:** Selected phase 1/2 trials in NTRK rearranged NSCLC

Trial	Clinical trials ID	Treatments	Objective response rate (%)	Median progression-free survival (months)	Median overall survival (months)
Navigate	NCT02568267	Larotrectinib	75	28.3	44.4
Pooled analysis of STARTRK-1, STARTRK-2, ALKA-372	NA	Entrectinib	70	14.9	NR

Selirectinib is a next generation TRK inhibitor with potent activity against secondary mutations. Among 31 larotrectinib pretreated patients, there was a 34% response rate, which rose to 45% when on-target resistance mutations, namely solvent-front mutations, were identified [239]. An early phase trial is ongoing (NCT03206931).^.^

Repotrectinib has high in vitro and in vivo efficacy against the solvent front TRKA^G595R^ and TRKC^G623R^ mutations [240] and a phase 1–2 trial is ongoing (NCT03093116). Similarly, the ROS1 and NTRK fusion next-generation inhibitor, talitrectinib, is currently in early phase trials (NCT02279433).

#### NRG1

In 2014, a neuregulin 1 (*NRG1*) fusion was identified for the first time in NSCLC, when five *CD74-NRG1* fusions were detected among invasive mucinous adenocarcinoma patients [241]. Subsequently, at least 16 additional fusion partners were identified, the most common being *SDC4* and *CD74*. Nonetheless, NRG1 fusions remain rare. Their incidence has been found to be of 0.2% among solid tumours, with roughly two thirds found in NSCLC, in which they are detected in 0.3% of samples [242]. Interestingly, while NRG1 is predominantly found in lung adenocarcinoma, particularly mucinous subtypes, 6% of cases are detected in squamous cell carcinoma [242, 243].

In mucinous adenocarcinoma, which account for about 5% of adenocarcinomas, until recently, only KRAS mutations were known to be enriched, detected in 50–80% of cases. *CD74-NRG1* fusions represent roughly 15% of this subtype [244]. NRG1 positive tumours are more common among non-smokers.

*NRG1* is located on chromosome 10 (10q23.1). It encodes a growth factor with structural similarity to HER receptor tyrosine kinases but from the heregulin protein family [243]. When the NRG1 receptor binds its ligand, it activates a HER2-HER3 heterocomplex that leads to PI3K-AKT-mTOR and MAPK signalling cascades, controlling cell proliferation, differentiation and survival [245].

The diagnosis of *NRG1* fusions relies on NGS with an RNA fusion panel [242]. These fusions are true oncogenic drivers and are mutually exclusive with EGFR and KRAS mutations and *ALK, ROS1* and *RET* rearrangements.

On a therapeutic level, the pan-HER TKI, afatinib, has demonstrated clinical activity in *NRG1* rearranged lung cancers with fusions including *SDC4-NRG1, SLC3A2-NRG1* and *CD74-NRG1*. In the real world eNRGy1 global registry, the efficacy of chemoimmunotherapy and immunotherapy was disappointing, with response rates of 0% and 20% and median progression-free survival rates of 3.3 months and 3.6 months, respectively. Afatinib demonstrated a 25% response rate, which was not contingent on the fusion partner, with a 2.8 month median progression-free survival [246]. Currently, afatinib is being explored prospectively in tumours with NRG1 fusions in the TAPUR (NCT02693535) and DRUP (NCT02925234) trials. Zenocutuzumab is a bispecific antibody targeting HER2 and HER3 preventing their combination in case of NRG1 fusion; it is currently assessed in a phase I/II trial including NSCLC with NRG fusion, with preliminary results showing a 35% ORR [247] (Table 11).

**Table 11:** Selected registry and phase 1/2 trials in NRG1 rearranged NSCLC

Trial	Clinical trials ID	Treatments	Objective response rate (%)	Median progression-free survival (months)	Median overall survival (months)
Schram et al	NCT02912949	Zenocotuzumab	35	NR	NR
Drilon et al	NA	Afatinib	25	2.8	NR

*Abbreviations*: *NSCLC* non-small cell lung cancer, *NRG1* neuregulin 1, *NR* not reported

### Potential targets

While the above are oncogenic drivers with effective therapies, some targets such as fibroblast growth factor receptor (FGFR) and PI3K alterations are less well understood.

### FGFR

FGFR is involved in cell proliferation, survival, dissemination and angiogenesis [248]. FGFR signalling dysregulation can be activated by ligand-dependent or independent mechanisms. The predominant causes include *FGFR* amplifications, mutations and translocations, which all can induce FGFR overexpression and constitutive tyrosine kinase activation [249].

Different cancer types harbour a distinct distribution of FGFR alterations. For instance, FGFR3 point mutations and less frequent *FGFR*3 rearrangements are found in 20% of advanced urothelial carcinomas, while *FGFR*2/3 fusions are of particular interest in cholangiocarcinomas, in which they occur in 14% of cases [250, 251]. In NSCLC, *FGFR*1 amplifications are found in roughly 20% of squamous cell carcinoma [252] and 3% of adenocarcinoma [253, 254].

*FGFR1*/3 fusions occur in 1% of patients with NSCLC, with a higher incidence in squamous histology, at around 3% [255]. Activating FGFR mutations, found in 4% of NSCLC, generally occur outside the kinase domain, unlike in EGFR, for example. They can lead to higher ligand-binding affinity or constitutive receptor dimerization [256].

FGFR1 is located on chromosome 8p12. Its amplification is associated with smoking history, while other clinical and demographic characteristics are not correlated with FGFR1. FGFR fusions are more common in patients who are smokers, with squamous lung cancer and poorly differentiated disease. In adenocarcinoma, they occur in 0.5% of diseases and are more frequent among non or light smokers [257, 258]. Most *FGFR3* fusions are with the TACC3 protein, transforming acidic coiled-coil containing protein 3.

Multikinase inhibitors appear ineffective in FGFR mutant solid tumours, with doses limited by hypertension caused by VEGFR inhibition. Similarly, selective inhibitors such as infigratinib, AZD4547 or BGJ398, targeting FRGR1 amplifications have been disappointing with response rates in squamous cell carcinoma under 10% [259, 260]. FGFR mRNA expression appears to be a superior predictive biomarker for FGFR-driven disease [261]. Recently, the highly-specific oral FGFR1-4 TKI, rogaratinib, was assessed in previously treated patients with squamous cell carcinoma and FGFR mRNA overexpression. The study failed, without any responses and a 1.6 month median progression-free survival [262]. Similarly, in a substudy of the Lung-MAP trial, the FGFR inhibitor, AZD4547, was assessed in previously treated patients with FGFR amplifications, mutations or fusions. The response rate was under 10% and median progression-free survival was 2.7 months [263].

In a database of over 57 000 advanced NSCLC patients, *FGFR2* and *FGFR3* fusions were detected in 0.02% and 0.26%, respectively. *FGFR3-TACC3* fusions account for over 91% of them, while fusions co-occurred with EGFR mutations in roughly 24% of cases. Among 3 patients with *FGFR3-TACC3* fusions as resistance mechanisms to EGFR TKIs, two were treated with an EGFR TKI with the FGFR TKI, erdafitinib, with a 6 and 13 month response [264]. These results highlight the complexity of FGFR alterations.

### PI3K

The PI3K/Akt/mTOR pathway is implicated in carcinogenesis and disease progression in NSCLC. It is a critical part of many signalling pathways of common oncogenic drivers. However, *PIK3CA* and amplifications and mutations are also frequently detected in NSCLC, in 3.7–19% of cases, with 2.9–6.2% in adenocarcinoma and 8.9–33% in squamous cell carcinoma [265, 266]. In one study, 57% of patients had concurrent EGFR, ALK, BRAF or KRAS oncogenic alterations [266]. *PIK3CA* alterations can be mechanisms of primary resistance or acquired resistance to other molecular oriented therapies. Primary *PIK3CA* alterations do not appear to have any prognostic influence, suggesting that they may be passenger alterations rather than true drivers of disease [266].

In lung cancer, trials have been largely disappointing. Pan‐class I PI3K inhibitors include pictilisib, PX-866, buparlisib and pilaralisib. Pictilisib failed to show a survival benefit with chemotherapy in the first-line setting, while PX‐866 failed with docetaxel in the second-line, though in molecularly unselected patients [267]. In the phase 2 BASALT‐1 trial, buparlisib was given to previously treated NSCLC patients with PI3K pathway alterations, but this and further buparlisib combination trials with chemotherapy or gefitinib, the latter in EGFR resistance, failed to show any efficacy [267–269].

Isoform specific PI3K inhibitors have also been studied. Alpelisib is the only currently approved PIK3CA inhibitor, used in advanced breast cancer. A phase 2 trial in NSCLC is ongoing for advanced diseases with *PIK3CA* mutations or amplifications [270]. Taselisib, a selective PI3K inhibitor, was evaluated in the phase 2 LUNG-MAP trial in previously treated patients with *PIK3CA* mutations, but this arm was closed for futility [271]. As such, given the high cutaneous toxicity, and lack of efficacy of current strategies, a greater understanding of PI3K targeting is required.

## Conclusion

The last decade has underscored the importance of molecular subtypes of NSCLC. Targeted therapies represent a paradigm shift, changing patients’ quality of life and survival prospects. Molecular testing in NSCLC is crucial. In this review, we highlighted the current therapeutic options, understanding of acquired resistance, and potential paths forwards. In spite of the phenomenal progress, we have experienced and the arsenal of therapies available to our patients today, there remain hurdles to overcome. Some targets are beginning to shift from undruggable to druggable, such as *KRAS* mutations, while others remain elusive, such as *PI3K* and *FGFR* alterations. As with FGFR, the first challenge with PI3K alterations will be to define criteria to determine cases of NSCLC where it is an oncogenic driver. The next hurdle will be successfully targeting it and to develop tolerable drugs.

For common alterations, we require a deeper understanding of mechanisms of resistance, in order to prevent and treat emerging acquired resistance. The arrival of liquid biopsies may further our understanding of this complex biology, by removing the invasive component of serial analyses and overcoming the limitation of sample heterogeneity in tumour tissue. Defining the best therapeutic sequence when several inhibitors are available also remains a challenge, with a tendency to use the most effective inhibitor in the first line to prevent the emergence of resistance.

What we can learn from the failures of precision oncology to-date is that there is more to targeting oncogenes than meets the eye. In clinical practice, identifying a potential driver should not lead to rogue therapy. The literature should be consulted, each case should be discussed in a molecular tumorboard and patients should be offered standard of care therapy before contemplating anything further. Whenever possible, patients should be included in clinical trials, as this is the only way other physicians will have answers. Clinical trials overcome the positive publication bias of case reports and may help future patients provide optimal care, either with targeted therapy or by avoiding unnecessary toxicity.

The ESMO Scale for Clinical Actionability of molecular Targets (ESCAT) was designed to assess clinical utility of oncogene-matched therapy. In breast cancer, the recent SAFIR02-Breast study randomized patients between targeted therapy and standard chemotherapy depending on genomic alterations. Only patients with ESCAT class I or II alterations derived a progression-free survival benefit from targeted therapy, molecular-matched therapies were wholly ineffective among patients with other alterations [272]. These ESCAT categories correspond to oncogenic drivers that have proven efficacy in prospective or retrospective trials [273] for the specific tumour type. A similar trial to that in breast cancer is ongoing in lung cancer, the SAFIR02-Lung study (NCT02117167).

Another trial of great importance is the Lung-MAP umbrella trial. This multicentric trial screens roughly 1000 patients with NSCLC each year for over 200 oncogenes. If one is detected, patients are be treated with molecular-matched therapy and data are collected. Some data have been published, including from a squamous cell carcinoma substudy, with negative results for FGFR and PI3K, discussed previously [274].

Trials such as these help tailor therapy and are the optimal approach for identifying the role of potential new molecular targets and their matched therapy. Furthermore, the treatment landscape is changing, as targeted therapies for EGFR have entered the non-metastatic setting, following the results of the ADAURA trial [275]. Further trials are assessing the impact of EGFR and ALK inhibitors in the non-metastatic setting and may provide new patterns of resistance. While precision oncology in NSCLC has made great strides, we still have much to learn.

## Data Availability

Not applicable.
